# Protocol for the CONNECT project: a mixed methods study investigating patient preferences for communication technology use in orthopaedic rehabilitation consultations

**DOI:** 10.1136/bmjopen-2019-035210

**Published:** 2019-12-11

**Authors:** Anthony William Gilbert, Jeremy Jones, Maria Stokes, Emmanouil Mentzakis, Carl R May

**Affiliations:** 1 Therapies Department, Royal National Orthopaedic Hospital Stanmore, Stanmore, Middlesex, UK; 2 Faculty of Health Sciences, University of Southampton, Southampton, UK; 3 Faculty of Economic, Social and Political Science, University of Southapton, Southampton, UK; 4 London School of Hygiene and Tropical Medicine Faculty of Epidemiology and Population Health, London, UK

**Keywords:** Adult orthopaedics, Musculoskeletal disorders, Telemedicine

## Abstract

**Introduction:**

Technology has been placed at the centre of global health policy and has been cited as having the potential to increase efficiency and remove geographical boundaries for patients to access care. Communication technology may support patients with orthopaedic problems, which is one of the leading causes of disability worldwide. There are several examples of technology being used in clinical research, although uptake in practice remains low. An understanding of patient preferences will support the design of a communication technology supported treatment pathway for patients undergoing orthopaedic rehabilitation.

**Methods and analysis:**

This mixed methods project will be conducted in four phases. In phase I, a systematic review of qualitative studies reporting communication technology use for orthopaedic rehabilitation will be conducted to devise a taxonomy of tasks patients’ face when using these technologies to access their care. In phase II, qualitative interviews will investigate how the work of being a patient changes during face-to-face and communication technology consultations and how these changes influence preference. In phase III, a discrete choice experiment will investigate the factors that influence preferences for the use of communication technology for orthopaedic rehabilitation consultations. Phase IV will be a practical application of these results. We will design a ‘minimally disruptive’ communication technology supported pathway for patients undergoing orthopaedic rehabilitation.

**Ethics and dissemination:**

The design of a pathway and underpinning patient preference will assist in understanding factors that might influence technology implementation for clinical care. This study requires ethical approval for phases II, III and IV. Approvals have been received for phase II (approval received on 4 December 2016 from the South Central-Oxford C Research Ethics Committee (IRAS ID: 255172, REC Reference 18/SC/0663)) and phase III (approval received on 18 October 2019 from the London-Hampstead Research Ethics Committee (IRAS ID: 248064, REC Reference 19/LO/1586)) and will be sought for phase IV. All participants will provide informed written consent prior to being enrolled onto the study.

**PROSPERO registration number:**

CRD42018100896.

**Strengths and limitations of this study**

- A taxonomy of patient ‘work’ and characterisation of patient preferences when using communication technology will assist in understanding implementation processes.
- This combination of sociological and economic research methods is novel: there are very few studies of patient preferences in telemedicine research.
- The design of a new consultation pathway, underpinned by patient preferences, may enhance the prospects of successful implementation in practice.
- This research is being conducted across two sites and may not be representative of the National Health Service nationwide.

## Introduction

Technology has been placed at the centre of global healthcare policy. Technology has been cited as having the potential to improve the effectiveness of healthcare systems through efficiency gain strategies^1^ and healthcare reform.^2^ Technology may overcome geographical boundaries^3^ with one example, from the Republic of Indonesia Health System Review,^1^ stating “A telemedicine network would enable patients in remote areas to have access to reliable medical consultations, and at the same time health professionals in remote areas can also be supported through the use of telemedicine technology”. In the UK, as outlined in the National Health Service (NHS) Long Term Plan,^4^ digital-first primary care will become a new option for every patient intending to provide fast access to convenient primary care with 95% of general practice patients to be offered e-consultation and other digital services in 2019.^5^

Musculoskeletal disease is the second largest cause of disability worldwide.^6^ It is widely accepted that the presence of osteoarthritis (OA) increases with age,^7^ although more than half of people with symptomatic OA are younger than 65.^8^ It is likely that many of these younger people will live for another 2–3 decades and require ongoing support and management that requires visits to healthcare practitioners. Communication technology, the use of technology to support the communication from a distance, is a digital innovation that can support patients to attend appointments.

There are several examples of communication technology to support the management of musculoskeletal disorders in the literature. The Virtual Outreach Project^9^ compared joint teleconsultations between hospital specialists, general practitioners and their patients in the UK and found the Virtual Outreach group to have significant increases in satisfaction compared with the face-to-face group. PhysioDirect^10^ telephone assessment was found to be as effective as face-to-face care for patients with musculoskeletal disorders accessing their care via phone. Skype, a free-to-access videoconferencing software, has been used across a range of clinical specialities.^11^ Greenhalgh’s VOCAL study^12^ found video outpatient consultations to be safe, effective and convenient in appropriate situations. Our previous research found the use of Skype videoconferencing for patients with shoulder instability to be acceptable for half of the patients.^13^ In our study, there were several factors that influenced patient’s choices between face-to-face and Skype consultations. We believe that further research on this area may assist with implementation of communication technology in clinical practice.

The process of implementing a new intervention (such as the introduction of communication technology in healthcare) has been demonstrated to be dependent on how the intervention is operationalised by its users,^14^ the ‘work’ people do when they implement a new intervention^15^ and the mobilisation of resources over time^16^ across different settings.^17^ Normalisation Process Theory (NPT) frames implementation processes through its focus on the things people *do* when they implement a new intervention in practice and provides the theoretical underpinning of phase I.

NPT has been used to determine the components of patient ‘work’ in chronic heart failure,^18^ stroke and diabetes,^19^ and chronic obstructive pulmonary disease and lung cancer.^20^ Patient work in heart failure includes the work of developing an understanding of treatments, interacting with others to organise care, attending appointments, taking medications, enacting lifestyle measures and appraising treatments. Burden of Treatment Theory (BOT)^21^ explains how the capacity for action interacts with the work that stems from healthcare. We are particularly interested in BOT across different situations of consultation and BOT provides the theoretical underpinning of phase II.

Minimally disruptive medicine (MDM)^22^ is an approach to healthcare that seeks to reduce the workload for the patient and caregiver. MDM seeks to advance patient goals for healthcare using effective care programmes designed and implemented in a manner that minimises the negative impact the care programme imposes on their lives.^23^ A ‘minimally disruptive’ orthopaedic rehabilitation consultation is a consultation that:

- Has minimal negative imposition on the patient’s life.
- Offers a reduce workload for the patient.
- Ensures healthcare professionals and care are accessible to the patient.

The Care in Orthopaedics, burdeN of treatmeNt and the Effect of Cummunication Technology (CONNECT) Project uses the aforementioned theories to understand:

1. The workload of being a patient when using communication technology (using NPT).
2. How the situational nature of a communication technology and face-to-face consultation influence burden of treatment (using BOT) and patient preferences.
3. Patient preference in relation face-to-face and communication technology consultations.
4. What a ‘minimally disruptive’ orthopaedic rehabilitation consultation looks like in practice (MDM).

### Population

Adults≥18 years of age with orthopaedic conditions participated in this study.

### Philosophical underpinnings

This study is set within the abduction paradigm.^24^ Abduction is the production of a hypothesis based on surprising evidence and, when following this approach, researchers seek to choose the ‘best’ among many alternatives. Abduction sits in the philosophical tradition of pragmatism, an ideology that supports the notion that a proposition is true when it works satisfactorily. Within the context of this research, one can make assumptions that ‘certain’ patients may prefer virtual appointments to face-to-face appointments (or vice versa).

We hypothesise that certain patients may indicate they prefer virtual appointments to face-to-face appointments (or vice versa). Large-scale data collection in phase III will support theorisation of preference in this study group. The purpose of the research is to develop satisfactory propositions, based on these data, to explain patient preferences and to design a minimally disruptive pathway based on these propositions.

### Overall aim

This study aimed to understand the patient preferences for the use of communication technology in orthopaedic rehabilitation consultations and design a ‘minimally disruptive’ consultation pathway based on these preferences.

### Health condition

Patients with orthopaedic problems were included in this study.

**Figure 1:**
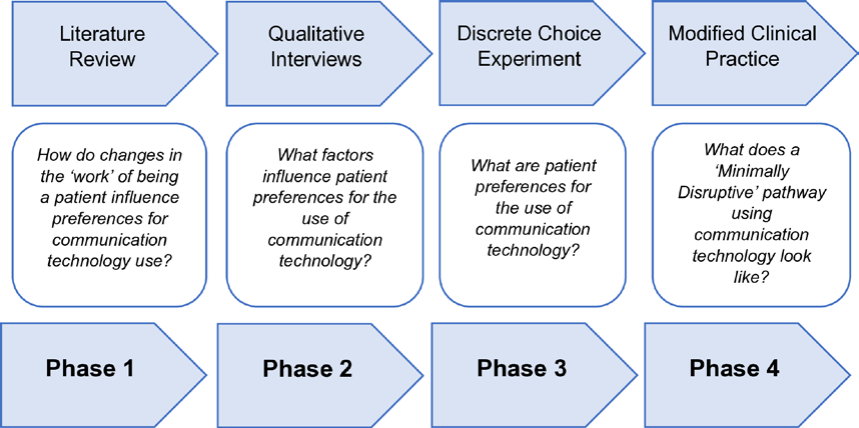
Overview of the CONNECT project.

An overview of the four phases of the CONNECT project is shown in figure 1.

#### Phase I: systematic review

We are interested in how the ‘work’ of being a patient influences preference. To the authors’ knowledge, no research has yet considered how the work of being a patient influences preference for communication technology consultations. The purpose of phase I is to develop a taxonomy of tasks required of patients using communication technology. We will then consider how factors relating to these tasks influence the comparative evaluation patients are faced when offered the choice of a communication technology or a face-to-face consultation for orthopaedic rehabilitation. This systematic review will be conducted using the Preferred Reporting Items for Systematic Reviews and Meta-Analyses (PRISMA) approach in order to answer the research question: *How do changes in the ‘work’ of being a patient when using communication technology influence patient preferences?* The protocol for this review was registered on the international prospective register of systematic reviews.^25^ The PRISMA Protocol (PRISMA-P) is demonstrated in online supplementary file 1.

10.1136/bmjopen-2019-035210.supp1 Supplementary data

MEDLINE, AMED, CINAHL, PsycINFO and SCOPUS will be searched from inception. The full search strategy, with search terms for each database, is available as a supplementary file (online supplementary file 2). Following the search, articles will be screened independently by two authors to identify full-text studies to be included in the review. A third author will be available to discuss any discrepancies.

10.1136/bmjopen-2019-035210.supp2 Supplementary data

Studies will be eligible for inclusion provided they meet the criteria for inclusion shown in table 1. Relevant studies will be first screened by their title and then by their abstract. The remaining texts will then read in full with all texts retained after this point for qualitative synthesis. Risk of bias will be screened using the Critical Appraisal Skills Programme (CASP) tool for qualitative studies.^26^ A discussion will be held between the authors to decide whether included studies are of sufficient quality to include in the review. A third author will be available to discuss any discrepancies. Reasons for exclusion will be listed.

**Table 1:** Eligibility criteria of studies

Inclusion	Exclusion
Full-text academic papers. Participants: Patients with an orthopaedic/musculoskeletal problem. Intervention: Studies reporting patients accessing physical assessment/rehabilitation using communication technology (eg, telephone, videoconferencing) in an orthopaedic/musculoskeletal setting. Outcome: Qualitative studies or studies with a qualitative component that focuses on the patient viewpoint of accessing communication technology.	Conference abstracts. Participants without an orthopaedic/musculoskeletal complaint. Quantitative studies. Studies not reporting patient viewpoints.

Full texts will be uploaded to QSR NVIVO software (QSR International Pty, V.12, 2018). NVIVO will be used to collect and organise data from the results, discussion and conclusion sections of each paper. Data will be collected by one author (AWG). For the purpose of data collection, the introduction and methods will be disregarded. The following process will then be followed:

1. Each sentence from the results, discussion and conclusion sections from the papers will be extracted and coded in NVIVO on a line-by-line basis. The codes will be attributed to each sentence based on their content.
2. An abductive analysis^24^ will then be conducted and will take three forms:
  - First, a thematic analysis of codes. This will enable authors to familiarise themselves with the content of the papers.
  - The following will be considered: *what is the work of being a patient when using communication technology?* Codes will then be organised into groups of codes depicting the *type* of work required of patients when using communication technology to access healthcare in order to develop a taxonomy of the types of work.
  - We will consider the question: *how might the work of being a patient when using communication technology influence patient preference?*
3. Data will be mapped out in the form of a model to demonstrate how, based on the included papers, the change in the ‘work’ of being a patient might influence preference for communication technology.

#### Phase II: qualitative interviews

Ethical approval received on 4 December 2016 from the South Central-Oxford C Research Ethics Committee (IRAS ID: 255172, REC Reference 18/SC/0663).

The aim of phase II is to explore how the use of communication technology changes the experience for patients receiving physiotherapy and occupational therapy for orthopaedic problems. This study will be conducted at one hospital. The results from phase II will frame the initial enquiry and interview schedule for phase II. Questions relating to Burden of Treatment Theory^21^ will explore the potential impact and workload changes for patients with the use of these technologies. The research question for phase II is: *How does communication technology use affect patient experience?* A focus on the circumstances in which patients would prefer to use communication technology will be used to inform the design of a discrete choice experiment (DCE) for phase III of the CONNECT project. These viewpoints (phase II) and the DCE (phase III) will inform the design of a modified clinical pathway (phase IV).

This study will use qualitative methodology to gain rich data regarding patient and clinicians’ opinions. Qualitative methods have been chosen to explore the underlying reasons behind these opinions. Semistructured interviews have been chosen to provide a loose guide and enable the researcher to explore pertinent themes relating to the research aims and objectives without the rigidity of a survey. The research paper reporting the results of phase II will be reported using the Standards for Reporting Qualitative Research (SRQR) checklist (the checklist for this protocol paper is available in online supplementary file 3).

10.1136/bmjopen-2019-035210.supp3 Supplementary data

The study will be conducted at one hospital site (a tertiary orthopaedic hospital). Participants will be recruited from the occupational and physiotherapy department of the hospital site. This study will aim to recruit 20 patients (five male, 5 female under the age of 49; 5 male, 5 female aged 50 and older) and 20 clinicians comprising physiotherapists and occupational therapists (at least 8 occupational therapists). This number has been selected to allow for a broad range of views within the scope and resources of a substudy within a PhD project. Patients are eligible for inclusion if they meet the inclusion criteria shown in table 2.

**Table 2:** Inclusion/exclusion criteria for phase II

Inclusion	Exclusion
Patients, over the age of 18 years, attending the hospital site for physiotherapy or occupational therapy. Patients who have experience of orthopaedic/musculoskeletal condition. Patients who are able to provide informed written consent to enter into the study. Patients able to understand and speak English or a language covered by the hospitals interpreter service	Patients without the capacity to consent. Patients suffering from disorders other than orthopaedic as the primary cause (eg, neurological or oncology disorders).

Participants who are eligible to enrol will be given a participant information sheet. All participants will have at least 24 hours to consider their participation and ask questions before being asked to provide informed, written consent. On receipt of consent, the participant will be recruited into the study. All participants will receive a copy of the consent form and a copy will also be saved in the project file. The lead researcher (AWG) is a practising physiotherapist at the hospital site. Patients will not be eligible for inclusion if they have previously, or are currently, been treated by AWG. At a mutually convenient time, the participants will be interviewed by AWG, either face-to-face or via video call using Skype or Zoom software. Interviews will be conducted using an interview guide developed on completion of phase I. All interviews will be audio recorded. All recordings will be linked anonymised using a unique study identifier, stored on an NHS password-encrypted computer and be sent off to an external company to be transcribed verbatim.

On receipt of the transcriptions, copies will be posted to all participants with an enclosed stamped addressed envelope. Participants will be given 2 weeks to review the transcriptions for factual accuracy and given the opportunity to add any additional comments. Transcripts will not be amended if the participant does not return them. At this stage, no other input will be required from research participants.

On receipt of amended transcripts or confirmation that no changes are required, transcripts will be uploaded into NVIVO software for organisation of data. Each sentence from the included sections will be coded in NVIVO on a line-by-line basis. The codes will be labelled using a description of the content of the respective sentence. Data analysis will take three forms: first, a thematic analysis of codes. This will enable researchers to familiarise themselves with the content of the interviews. For the second iteration of coding, the following will be considered: *what is the work of being a patient when using communication technology?* Codes will then be organised into groups of codes depicting the *type* of work required of patients when using communication technology to access healthcare in order to develop a taxonomy of the types of work. The coding will be completed in a way that looks to extend the model in phase I. Throughout this process, we will consider the question: *how might the work of being a patient when using communication technology influence patient preference?* Specific data to support the design and development of future components of the CONNECT project (namely, phase III) will be organised separately.

#### Phase III: discrete choice experiment

Ethical approval was received on 18 October 2019 from the London-Hampstead Research Ethics Committee (IRAS ID: 248064, REC Reference 19/LO/1586).

The aim of phase III is to understand the factors that influence patient preference when presented with the choice between a face-to-face and communication technology consultation for orthopaedic rehabilitation. The research question for phase III is *what factors influence preferences for patients undergoing orthopaedic rehabilitation who are offered a face-to-face or communication technology consultation?*

The results from phase I and phase II will inform the design of the DCE. It is not clear at this stage what the attributes and individual levels will be. However, they are likely to include travel time and cost, perceived ease of use of equipment to engage in the consultation, raining requirements, conduct and content of the consultation and the number of engagements with clinicians during any given pathway.

A D-efficient design will be created in NGene software (Choice Metrics) where attribute non-linearity will be allowed (ie, levels of specific attributes allowed to have non-linear effects). To reduce cognitive burden on participants, the maximum number of choice sets will be limited to 12 and blocking will be used if required (ie, blocking implies orthogonally splitting the number choice sets into two or more groups which are then presented to different individuals).

This study will be conducted across two hospital sites (a tertiary orthopaedic hospital and a secondary care orthopaedic hospital). Participants will be recruited from the occupational and physiotherapy department of the hospital site. This study will aim to recruit at least 200 patients per site. Patients are eligible for inclusion if they meet the inclusion criteria shown in table 3.

**Table 3:** Inclusion/exclusion criteria for phase III

Inclusion	Exclusion
Patients, over the age of 18 years, attending either hospital site for physiotherapy or occupational therapy. Patients who have experience of orthopaedic/musculoskeletal condition. Patients who are able to provide informed written consent to enter into the study. Patients able to understand and speak English or a language covered by the hospitals interpreter service.	Patients without the capacity to consent. Patients suffering from disorders other than orthopaedic as the primary cause (eg, neurological or oncology disorders).

It is anticipated that around 200 participants per site will be recruited but precise numbers will be dependent on a power analysis once the number of questions and blocks has been ascertained. Participants who are eligible to enrol will be given a participant information sheet. All participants will be asked to provide informed, written consent at that time. On receipt of consent, the participant will be recruited into the study. All participants will receive a copy of the consent form and a copy will also be saved in the project file.

The DCE questionnaire will be designed using online questionnaire software (SurveyMonkey). The DCE will be administered in the choice of two forms—paper or electronically using a tablet computer—and patients will be offered the choice of completing at the study site or at home within 24 hours. All participants will be provided with an envelope to return the completed DCE questionnaire. Data from paper questionnaires will be manually entered by the researcher. Online SurveyMonkey questionnaires automatically exports data into Microsoft Excel. The initial questionnaire will be piloted on approximately 10 patients. This will undergo repeat piloting on further iterations of the DCE until the final design is established.

Initial reporting will provide descriptive data for demographic variables and observed choices (virtual vs face-to-face) by choice set. Given the binary set-up of the experiment, analysis will proceed with conditional logit and random parameter binary models.^27^ Attribute levels will enter as covariates to explain individual choices, while individual specific characteristics will either enter as interactions with attribute or directly, depending on the estimation model used. Following standard literature, unobserved heterogeneity, if present, will be explored through a random coefficient model. Trade-offs and marginal rates of substitution between attribute level will be calculated, while willingness-to-pay values will also be computed if cost is present in the final list of attributes for the experiment. The resulting factors that influence preferences will be used to further develop the model of patient preference from phases I and II.

#### Phase IV: pathway design

The aim of phase IV is to design a model of care based on the results from phases I–III. The research question for this phase is *what does a minimally disruptive consultation look like in orthopaedic rehabilitation?* A theoretical model of care developed during the results of phases I–III will be applied practically to orthopaedic rehabilitation. The model of rehabilitation will be designed and piloted on a small number of patients (approximately 10) and their clinicians to understand the impact of the new consultation format. A small study of acceptability will be conducted with a view to inform the further development of the model of care and to gain insight into the issues that might influence further upscale and transportability of the model of care in other settings. Ethical and Health Research Authority approval will be sought prior to commencing this phase.

#### Potential benefits to patients and to the NHS

Previous studies into the introduction of e-health technologies have used top-down models in which the methodologies and interventions have been decided by investigators without a complete understanding of patient preferences. These studies, although pointing to the value of e-health technologies, have not always led to routine uptake in clinical practice. The CONNECT project investigates the role of patient preferences in normalisation processes, and it is postulated that the knowledge of such patient preferences is more likely to lead to successful e-health implementation. This project will focus on orthopaedic rehabilitation appointments, but it will have much wider implications for the introduction of e-health technology to other spheres of medicine. There is the potential both to provide a better patient service and to effect cost savings to society and the healthcare system.

### Patient and public involvement

The CONNECT Project Patient and Public Involvement steering group (PPISG) has been set up to provide guidance on the conduct of the research (details available from www.theconnectproject.info). The first meeting of the PPISG was held in August 2016 prior to the submission of the research to the National Institute for Health Research in May 2017. A discussion was held about the overall research aims which supported the identification of the research questions. The PPISG has supported the design of the overall research plan and will continue to be involved during the development and refinement of each phase prior to the completion of each study protocol. The participant information and consent forms and the discrete choice experiement questionnaire for phase III has been reviewed by the PPISG. In addition, the PPISG will support the development of the lay summary outputs to be disseminated to patients and the public.

### Ethics and dissemination

The design of a pathway and underpinning patient preference will assist in understanding factors that might influence technology implementation for clinical care. This study requires ethical approval for phases II, III and IV. Approvals have been received for phase II (approval received on 4 December 2016 from the South Central-Oxford C Research Ethics Committee (IRAS ID: 255172, REC Reference 18/SC/0663)) and phase III (approval received on 18 October 2019 from the London-Hampstead Research Ethics Committee (IRAS ID: 248064, REC Reference 19/LO/1586)) and will be sought for phase IV. All participants will provide informed written consent prior to being enrolled onto the study.

A manuscript will be written for publication for each phase and submitted to national and international conferences. In addition, lay summary results will be developed and made available for patients and the public. All results will be published in open access peer-reviewed journals. Links to research outputs will be made available on the CONNECT project website available at www.theconnectproject.info.

## Supplementary Material

Reviewer comments

Author's manuscript
