# Supplementary material for: Protocol for the CONNECT project: a mixed methods study investigating patient preferences for communication technology use in orthopaedic rehabilitation consultations
Source: BMJ Open. 2019 Dec 11;9(12):e035210. doi: 10.1136/bmjopen-2019-035210 (PMC6924859; doi:10.1136/bmjopen-2019-035210)
Supplement: Supplementary data [file bmjopen-2019-035210supp002.pdf]

The CONNECT Project Phase 1 Search Strategy

Searches

English Language studies will be searched for with no date restrictions. The following databases will be searched using a combination of keywords and database specific subject headings: MEDLINE, AMED, CINAHL, PsycINFO and Scopus. Forward and backwards reference screening of identified papers and relevant systematic reviews will be completed using Web of Science.

Types of study to be included

Qualitative studies or studies with a qualitative component (use of interviews or focus groups)

Condition or domain being studied

Orthopaedics and musculoskeletal pathologies

Participants/population

Inclusion:
• Full text academic papers.
• Patients with an orthopaedic / musculosketal problem
• Studies reporting patients accessing clinical care (assessment / rehabilitation) through the use of real time communication technology (eg telephone, videoconferencing) in an orthopaedic / musculoskeletal setting.
• Qualitative studies or studies with a qualitative component (use of interviews or focus groups)

Exclusion:
• Conference abstracts
• Participants without an orthopaedic / musculoskeletal complaint
• Quantitative studies
• Studies not reporting patient viewpoints

Intervention(s), exposure(s)

Inclusion: Participants accessing clinical care (assessment / rehabilitation) through the use of communication technology in real time (eg telephone, videoconferencing) in an orthopaedic / musculoskeletal setting.

Comparator(s)/control

N/A

Context

Studies will be included providing they report the perspectives of patients regarding access to musculoskeletal / orthopaedic assessment / rehabilitation. Studies may also contain perspectives of clinicians in addition to the viewpoints of patients.

Main outcome(s)

Patient experience and workload of using communication technology to access musculoskeletal / orthopaedic assessment / rehabilitation.

Timing and effect measures

Not applicable.

Additional outcome(s)

None

Timing and effect measures

None

Data extraction (selection and coding)

Two researchers will independently identify full text papers meeting the inclusion criteria. Discrepancies arising regarding selection will be resolved through discussion with a third researcher.

Risk of bias (quality) assessment

Two researchers will independently screen the full text included studies for risk of bias. The Critical Appraisal Skills Programme Qualitative Tool will be used. Discrepancies arising regarding risk of bias will be resolved through discussion with a third researcher.

Strategy for data synthesis

All studies included for full text review will be analysed. Data extracted from the Methods, Results, Discussion and Conclusion sections will be analysed by using an attribution analysis. A taxonomy of attributions will be thematically analysed using NVIVO software in accordance with Normalisation Process Theory.

Search Terms

Database	Search Term
MEDLINE	(communication technology OR e-health OR telecare OR telemedicine OR telehealth OR telemonitoring OR videoconferencing OR real time videoconferencing OR real time 1:1 videoconferencing OR telecommunication OR virtual OR (MH "telemedicine") OR (MH "remote consultation") OR (MH "videoconferencing")) AND (Focus group OR Focus groups OR observation OR Ethnography OR Ethnographic OR Phenomenology OR Phenomenological OR Lived experience OR Grounded theory OR Thematic analysis OR Conversation analysis

	OR Framework analysis OR (MH "Qualitative Research")) AND (physio OR physiotherapy OR physical therapy OR rehab OR rehabilitation)
AMED	(communication technology OR e-health OR telecare OR telemedicine OR telehealth OR telemonitoring OR videoconferencing OR real time videoconferencing OR real time 1:1 videoconferencing OR telecommunication OR virtual OR (SU (telemedicine) OR (SU (technology medical))) AND (Focus group OR Focus groups OR observation OR Ethnography OR Ethnographic OR Phenomenology OR Phenomenological OR Lived experience OR Grounded theory OR Thematic analysis OR Conversation analysis OR Framework analysis) AND (physio OR physiotherapy OR physical therapy OR rehab OR rehabilitation)
CINAHL	(communication technology OR e-health OR telecare OR telemedicine OR telehealth OR telemonitoring OR videoconferencing OR real time videoconferencing OR real time 1:1 videoconferencing OR telecommunication OR virtual OR (MH “Videoconferencing”) OR (MH “Remote Consultation”)) AND (Focus group OR Focus groups OR observation OR Ethnography OR Ethnographic OR Phenomenology OR Phenomenological OR Lived experience OR Grounded theory OR Thematic analysis OR Conversation analysis OR Framework analysis OR (MH “qualitative studies”)) AND (physio OR physiotherapy OR physical therapy OR rehab OR rehabilitation)
PsychINFO	(communication technology OR e-health OR telecare OR telemedicine OR telehealth OR telemonitoring OR videoconferencing OR real time videoconferencing OR real time 1:1 videoconferencing OR telecommunication OR virtual OR (DE "Telemedicine")) AND (Focus group OR Focus groups OR observation OR Ethnography OR Ethnographic OR Phenomenology OR Phenomenological OR Lived experience OR Grounded theory OR Thematic analysis OR Conversation analysis OR Framework analysis OR DE "Qualitative Research")) AND (physio OR physiotherapy OR physical therapy OR rehab OR rehabilitation)

SCOPUS	1. (TITLE-ABS-KEY (“communication technology” OR e-health OR telecare OR telemedicine OR telehealth OR telemonitoring OR videoconferencing OR “real time videoconferencing” OR “real time 1:1 videoconferencing” OR telecommunication OR virtual))<br>2. (TITLE-ABS-KEY (“Focus group” OR “Focus groups” OR observation OR Ethnography OR Ethnographic OR Phenomenology OR Phenomenological OR “Lived experience” OR “Grounded theory” OR “Thematic analysis” OR “Conversation analysis” OR “Framework analysis”))<br>3. (TITLE-ABS-KEY (physio OR physiotherapy OR physical therapy OR rehab OR rehabilitation)<br>4. #1 AND #2 AND #3
