# Supplementary material for: Protocol for the CONNECT project: a mixed methods study investigating patient preferences for communication technology use in orthopaedic rehabilitation consultations
Source: BMJ Open. 2019 Dec 11;9(12):e035210. doi: 10.1136/bmjopen-2019-035210 (PMC6924859; doi:10.1136/bmjopen-2019-035210)
Supplement: Author's manuscript [file bmjopen-2019-035210.draft_revisions.pdf]

BMJ Open is committed to open peer review. As part of this commitment we make the peer review history of every article we publish publicly available.

When an article is published we post the peer reviewers' comments and the authors' responses online. We also post the versions of the paper that were used during peer review. These are the versions that the peer review comments apply to.

The versions of the paper that follow are the versions that were submitted during the peer review process. They are not the versions of record or the final published versions. They should not be cited or distributed as the published version of this manuscript.

BMJ Open is an open access journal and the full, final, typeset and author-corrected version of record of the manuscript is available on our site with no access controls, subscription charges or pay-per-view fees (http://bmjopen.bmj.com).

If you have any questions on BMJ Open's open peer review process please email info.bmjopen@bmj.com

Patient preferences for communication technology use in orthopaedic rehabilitation consultations: Protocol for the CONNECT Project

Journal:	*BMJ Open*
Manuscript ID	bmjopen-2019-035210
Article Type:	Protocol
Date Submitted by the Author:	23-Oct-2019

Complete List of Authors:	Gilbert, Anthony; Royal National Orthopaedic Hospital Stanmore, Therapies Department; University of Southampton, Faculty of Health Sciences<br>Jones, Jeremy; University of Southampton, Faculty of Health Sciences<br>Stokes, Maria; University of Southampton, Faculty of Health Sciences<br>Mentzakis, Emmanouil; University of Southapton, Faculty of Economic, Social and Political Science<br>May, Carl; London School of Hygiene and Tropical Medicine Faculty of Epidemiology and Population Health

Keywords:	Adult orthopaedics < ORTHOPAEDIC & TRAUMA SURGERY, Musculoskeletal disorders < ORTHOPAEDIC & TRAUMA SURGERY, Telemedicine < BIOTECHNOLOGY & BIOINFORMATICS

SCHOLARONE™
Manuscripts

Patient preferences for communication technology use in orthopaedic rehabilitation consultations: Protocol for the CONNECT Project

Authors

1. Anthony Gilbert[1, 2]
2. Jeremy Jones[2]
3. Maria Stokes[2]
4. Emmanouil Mentzakis[3]
5. Carl R May[4]

Author Affiliations

1. Therapies Department, Royal National Orthopaedic Hospital, Stanmore, UK

2. School of Health Sciences, Faculty of Environment and Life Sciences, University of Southampton, Southampton, UK

3. Faculty of Economic, Social and Political Science, University of Southampton, Southampton, UK

4. Faculty of Public Health and Policy, London School of Hygiene and Tropical Medicine, London, UK

Corresponding author: Anthony Gilbert anthony.gilbert@nhs.net

For peer review only

For peer review only - http://bmjopen.bmj.com/site/about/guidelines.xhtml

Abstract

Introduction

Technology has been placed at the centre of global health policy and has been cited as having the potential to increase efficiency and remove geographical boundaries for patients to access care. Communication technology may support patients with orthopaedic problems which is one of the leading causes of disability worldwide. There are several examples of technology being used in clinical research although uptake in practice remains low. An understanding of patient preferences will support the design of a communication technology supported treatment pathway for patients undergoing orthopaedic rehabilitation.

Methods and Analysis

This mixed methods project will be conducted in four phases. In phase 1 a systematic review of qualitative studies reporting communication technology use for orthopaedic rehabilitation will be conducted to devise a taxonomy of tasks patients face when using these technologies to access their care. In phase 2 qualitative interviews will investigate how the work of being a patient changes during face-to-face and communication technology consultations and how these changes influence preference. In Phase 3 a Discrete Choice Experiment will investigate the factors that influence preferences for the use of communication technology for orthopaedic rehabilitation consultations. Phase 4 will be a practical application of these results. We will design a 'minimally disruptive' communication technology supported pathway for patients undergoing orthopaedic rehabilitation.

Registration

PROSPERO registration number: ID=CRD42018100896.

For peer review only

For peer review only - http://bmjopen.bmj.com/site/about/guidelines.xhtml

Strengths and Limitations of this Study

- A taxonomy of patient ‘work’ and characterization of patient preferences when using communication technology will assist in understanding implementation processes.
- This combination of sociological and economic research methods is novel: there are very few studies of patient preferences in telemedicine research.
- The design of a new consultation pathway, underpinned by patient preferences, may enhance the prospects of successful implementation in practice.
- This research is being conducted across two sites and may not be representative of the NHS nationwide.

For peer review only

For peer review only - http://bmjopen.bmj.com/site/about/guidelines.xhtml

Introduction

Technology has been placed at the center of global healthcare policy. Technology has been cited as having the potential to improve the effectiveness of healthcare systems through efficiency gain strategies[1] and healthcare reform[2]. Technology may overcome geographical boundaries[3] with one example, from the Republic of Indonesia Health System Review[1], stating 'A telemedicine network would enable patients in remote areas to have access to reliable medical consultations, and at the same time health professionals in remote areas can also be supported through the use of telemedicine technology'. In the United Kingdom, as outlined in the NHS Long Term Plan[4], digital-first primary care will become a new option for every patient intending to provide fast access to convenient primary care with 95% of GP patients to be offered e-consultation and other digital services in 2019[5].

Musculoskeletal disease is the second largest cause of disability worldwide[6]. It is widely accepted that the presence of osteoarthritis (OA) increases with age[7] although more than half of people with symptomatic OA are younger than 65[8]. It is likely that many of these younger people will live for another 2-3 decades and require ongoing support and management that requires visits to healthcare practitioners. Communication technology, the use of technology to support the communication from a distance, is a digital innovation that can support patients to attend appointments.

There are several examples of communication technology to support the management of musculoskeletal disorders in the literature. The Virtual Outreach Project[9] compared joint teleconsultations between hospital specialists, General Practitioners and their patients in the United Kingdom and found the Virtual Outreach group to have significant increases in satisfaction compared to the face-to-face group. PhysioDirect[10] telephone assessment was found to be as effective as face-to-face care for patients with musculoskeletal disorders accessing their care via phone. Skype, a free-to-access videoconferencing software, has been used across a range of clinical specialities[11]. Greenhalgh's VOCAL study[12] found video outpatient consultations to be safe, effective and convenient in appropriate situations. Our previous research found the use of Skype videoconferencing for patients with shoulder instability to be acceptable for half of the patients[13]. In our study, there were several factors that influenced patient's choices between face-to-face and Skype consultations. We believe that further research on this area may assist with implementation of communication technology in clinical practice.

The process of implementing a new intervention (such as the introduction of communication technology in healthcare) has been demonstrated to be dependent on how the intervention is operationalised by its users[14], the "work" people do when they implement a new intervention[15], the mobilisation of resources over time[16] across different settings[17]. Normalisation Process Theory (NPT) frames implementation processes through its focus on the things people *do* when they implement a new intervention in practice and provides the theoretical underpinning of phase 1.

For peer review only - http://bmjopen.bmj.com/site/about/guidelines.xhtml

NPT has been used to determine the components of patient "work" in chronic heart failure[18], stroke and diabetes[19] and Chronic Obstructive Pulmonary Disease and lung cancer[20]. Patient work in heart failure includes the work of developing an understanding of treatments, interacting with others to organise care, attending appointments, taking medications, enacting lifestyle measures and appraising treatments. Burden of treatment theory (BOT)[21] explains how the capacity for action interacts with the work that stems from healthcare. We are particularly interested in BOT across different situation of consultation and BOT provides the theoretical underpinning of phase 2.

Minimally Disruptive Medicine (MDM)[22] is an approach to healthcare that seeks to reduce the workload for the patient and caregiver. MDM seeks to advance patient goals for healthcare using effective care programmes designed and implemented in a manner that minimises the negative impact the care programme imposes on their lives[23]. A 'minimally disruptive' orthopaedic rehabilitation consultation is a consultation that:

- has minimal negative imposition on the patient's life
- offers a reduce workload for the patient
- ensures healthcare professionals and care are accessible to the patient

The CONNECT Project utilises the aforementioned theories to understand:

(i) the workload of being a patient when using communication technology (using NPT);
(ii) how the situational nature of a communication technology and face-to-face consultation influence burden of treatment (using BOT) and patient preferences;
(iii) patient preference in relation face to face and communication technology consultations;
(iv) what a 'minimally disruptive' orthopaedic rehabilitation consultation looks like in practice (MDM).

Population

Adults ≥ 18 years of age with orthopaedic conditions.

Philosophical Underpinnings

This study is set within the abduction paradigm[24]. Abduction is the production of a hypothesis based on surprising evidence and, when following this approach, researchers seek to choose the 'best' amongst many alternatives. Abduction sits in the philosophical tradition of pragmatism, an ideology that supports the notion that a proposition is true when it works satisfactorily. Within the context of this research, one can make assumptions that 'certain' patients may prefer virtual appointments to face-to-face appointments (or vice versa).

We hypothesise that certain patients may indicate they prefer virtual appointments to face-to-face appointments (or vice versa). Large scale data collection in phase 3 will support theorisation of preference in this study group. The purpose of the research is to develop satisfactory propositions, based on these data, to explain patient preferences and to design a minimally disruptive pathway based on these propositions.

Overall Aim

To understand the patient preferences for the use of communication technology in orthopaedic rehabilitation consultations and design a 'minimally disruptive' consultation pathway based on these preferences.

For peer review only - http://bmjopen.bmj.com/site/about/guidelines.xhtml

Health Condition

Patients with orthopaedic problems.

Methods & Analysis

An overview of the four phases of the CONNECT Project is shown in Figure 1.

Phase 1: Systematic Review

We are interested in how the 'work' of being a patient influences preference. The purpose of phase 1 is to develop a taxonomy of tasks required of patients using communication technology. We will then consider how factors relating to these tasks influence the comparative evaluation patients are faced when offered the choice of a communication technology or a face-to-face consultation for orthopaedic rehabilitation. This systematic review will be conducted using the PRISMA approach in order to answer the research question: *How do changes in the 'work' of being a patient when using communication technology influence patient preferences?* The protocol for this review was registered on the International prospective register of systematic reviews (PROSPERO registration number: ID=CRD42018100896)[25].

MEDLINE, AMED, CINAHL, PsychINFO and SCOPUS will be searched from inception. Full search terms and the search strategy is available to view in the review protocol. Articles will be screened independently by two authors with a third author available to discuss any discrepancies.

Studies will be eligible for inclusion providing they meet the criteria for inclusion shown in Table 1. Relevant studies will be firstly screened by their title and then by their abstract. Remaining texts will then read in full with all texts retained after this point for qualitative synthesis. Risk of bias will be screened using the CASP tool for qualitative studies[26]. A discussion will be held between two authors with a third author available to discuss any discrepancies, to decide whether included studies were of sufficient quality to include in the review.

Table 1: Eligibility Criteria of Studies

Inclusion:	Exclusion:
• Full text academic papers.<br>• Patients with an orthopaedic / musculoskeletal problem<br>• Studies reporting patients accessing physical assessment / rehabilitation using communication technology (e.g. telephone, videoconferencing) in an orthopaedic / musculoskeletal setting.<br>• Qualitative studies or studies with a qualitative component that focuses on the patient viewpoint of accessing communication technology.	• Conference abstracts<br>• Participants without an orthopaedic / musculoskeletal complaint<br>• Quantitative studies<br>• Studies not reporting patient viewpoints

Full texts will be uploaded to QSR NVIVO Software (QSR International Pty Ltd. Version 12, 2018). NVIVO will be used to collect and organize data from the results, discussion and conclusion sections of each paper. Each sentence from the included sections will be coded in NVIVO on a line by line basis. The codes will be labelled using a description of the content of the respective sentence. An Abductive Analysis[24] will be conducted and will take three forms: firstly, a thematic analysis of codes.

For peer review only - http://bmjopen.bmj.com/site/about/guidelines.xhtml

This will enable authors to familiarise themselves with the content of the papers. For the second iteration of coding the following will be considered: *what is the work of being a patient when using communication technology?* Codes will then be organised into groups of codes depicting the *type* of work required of patients when using communication technology to access healthcare in order to develop a taxonomy of the types of work. Following this, we will consider the question: *how might the work of being a patient when using communication technology influence patient preference?* Data will be mapped out in the form of a model to demonstrate how, based on the included papers, the change in the 'work' of being a patient might influence preference for communication technology.

Phase 2: Qualitative Interviews

Ethical Approval received 4th December 2016 from the South Central-Oxford C Research Ethics Committee (IRAS ID: 255172, REC Reference 18/SC/0663).

The aim of phase 2 is to explore how the use of communication technology changes the experience for patients receiving physiotherapy and occupational therapy for orthopaedic problems. This study will be conducted at one hospital. The results from phase 1 will frame the initial enquiry and interview schedule for phase 2. Questions relating to Burden of Treatment Theory[21] will explore the potential impact and workload changes for patients with the use of these technologies. The research question for phase 2 is: *How does communication technology use affect patient experience?* A focus on the circumstances in which patients would prefer to use communication technology will be used to inform the design of a Discrete Choice Experiment (DCE) for phase 3 of the CONNECT Project. These viewpoints (Phase 2) and the DCE (Phase 3) will inform the design of a modified clinical pathway (Phase 4).

This study will use qualitative methodology to gain rich data regarding patient and clinicians' opinions. Qualitative methods have been chosen to explore the underlying reasons behind these opinions. Semi structured interviews have been chosen to provide a loose guide and enable the researcher to explore pertinent themes relating to the research aims and objectives without the rigidity of a survey. This study will aim to recruit 20 patients (5 males, 5 females under the age of 49; 5 males, 5 females aged 50 and over) and 20 clinicians comprising of physiotherapists and occupational therapists (at least 8 occupational therapists).

Participants who are eligible to enrol will be given a participant information sheet. All participants will have at least 24 hours to consider their participation and ask questions before being asked to provide informed, written consent. Upon receipt of consent the participant will be recruited into the study. All participants will receive a copy of the consent form and a copy will also be saved in the project file. At a mutually convenient time, the participants will be interviewed by the lead researcher (AG), either face-to-face or via video call using Skype or Zoom software. Interviews will be conducted using an interview guide developed upon completion of phase 1. All interviews will be audio recorded. All recordings will be linked anonymised using a unique study identifier, stored on an NHS Password encrypted computer and be sent off to an external company to be transcribed verbatim.

Upon receipt of the transcriptions, copies will be posted to all participants with an enclosed stamped addressed envelope. Participants will be given two weeks to review the transcriptions for factual accuracy and given the opportunity to add any additional comments. Transcripts will not be amended if the participant does not return them. At this stage no other input will be required from research participants.

For peer review only - http://bmjopen.bmj.com/site/about/guidelines.xhtml

Upon receipt of amended transcripts or confirmation that no changes are required, transcripts will be uploaded into NVIVO software for organisation of data. Each sentence from the included sections will be coded in NVIVO on a line by line basis. The codes will be labelled using a description of the content of the respective sentence. Data analysis will take three forms: firstly, a thematic analysis of codes. This will enable authors to familiarise themselves with the content of the interviews. For the second iteration of coding the following will be considered: *what is the work of being a patient when using communication technology?* Codes will then be organised into groups of codes depicting the *type* of work required of patients when using communication technology to access healthcare in order to develop a taxonomy of the types of work. The coding will be completed in a way that looks to extend the model in phase 1. Throughout this process, we will consider the question: *how might the work of being a patient when using communication technology influence patient preference?* Specific data to support the design and development of future components of the CONNECT Project (namely phase 3) will be organised separately.

Phase 3: Discrete Choice Experiment

Ethical Approval received 18th October 2019 from the London-Hampstead Research Ethics Committee (IRAS ID: 248064, REC Reference 19/LO/1586).

The aim of phase 3 is to understand the factors that influence patient preference when presented with the choice between a face-to-face and communication technology consultation for orthopaedic rehabilitation. The research question for phase 3 is *what factors influence preferences for patients undergoing orthopaedic rehabilitation who are offered a face-to-face or communication technology consultation?*

This study will be conducted across two sites. The results from phase 1 and phase 2 will inform the design of the DCE. It is not clear at this stage what the attributes and individual levels will be. However, they are likely to include travel time and cost, perceived ease of use of equipment to engage in the consultation, raining requirements, conduct and content of the consultation and the number of engagements with clinicians during any given pathway.

A D-efficient design will be created in NGene software (Choice Metrics) where attribute non-linearity will be allowed (i.e. levels of specific attributes allowed to have non-linear effects). To reduce cognitive burden on participants the maximum number of choice sets will be limited to 12 and blocking will be utilized if required (i.e. blocking implies orthogonally splitting the number choice sets into two or more groups which are then presented to different individuals). It is anticipated that around 300 participants will be recruited but precise numbers will be dependent on a power analysis once the number of questions and blocks has been ascertained.

The DCE questionnaire will be designed using online questionnaire software (Survey Monkey). The DCE will be administered in the choice of 2 forms - paper or electronically using a tablet computer – and patients will be offered the choice of completing at the study site or at home within 24 hours. All patients will be provided with an envelope to return the completed DCE questionnaire. Data from paper questionnaires will be manually entered by the researcher. Online SurveyMonkey questionnaires automatically exports data into Microsoft Excel. The initial questionnaire will be piloted on approximately 10 patients. This will undergo repeat piloting on further iterations of the DCE until the final design is established.

Initial reporting will provide descriptive data for demographic variables and observed choices (virtual vs face-to-face) by choice set. Given the binary set-up of the experiment, analysis will proceed with conditional logit and random parameter binary models[27]. Attribute levels will enter as covariates to

For peer review only - http://bmjopen.bmj.com/site/about/guidelines.xhtml

explain individual choices, while individual specific characteristics will either enter as interactions with attribute or directly, depending on the estimation model used. Following standard literature, unobserved heterogeneity, if present, will be explored through a random coefficient model. Trade-offs and marginal rates of substitution between attribute level will be calculated, while willingness-to-pay values will also be computed if cost is present in the final list of attributes for the experiment. The resulting factors that influence preferences will be used to further develop the model of patient preference from phases 1 & 2.

Phase 4: Pathway Design

The aim of phase 4 is to design a model of care based on the results from phase 1-3. The research question for this phase is *what does a minimally disruptive consultation look like in orthopaedic rehabilitation?* A theoretical model of care developed during the results of phases 1-3 will be applied practically to orthopaedic rehabilitation. The model of rehabilitation will be designed and piloted on a small number of patients (approximately 10) and their clinicians to understand the impact of the new consultation format. A small study of acceptability will be conducted with a view to inform the further development of the model of care and to gain insight into the issues that might influence further upscale and transportability of the model of care in other settings.

Potential benefits to patients and to the NHS

Previous studies into the introduction of e-health technologies have used top down models in which the methodologies and interventions have been decided by investigators without a complete understanding of patient preferences. These studies, although pointing to the value of e-health technologies, have not always led to routine uptake in clinical practice. The CONNECT Project investigates the role of patient preferences in normalization processes, and it is postulated that the knowledge of such patient preferences is more likely to lead to successful e-health implementation. This project will focus on orthopaedic rehabilitation appointments, but it will have much wider implications for the introduction of e-health technology to other spheres of medicine. There is the potential to both provide a better patient service and to effect cost savings to society and the healthcare system.

Patient and Public Involvement

The CONNECT Project Patient and Public Involvement steering group (PPISG) has been set up to provide guidance on the conduct of the research (details available from www.theconnectproject.info). The CONNECT Project PPISG has supported the design of the overall research plan and will be involved during the development of each phase prior to the completion of each study protocol. The CONNECT PPISG will support the development of the lay-summary outputs to be disseminated to patients and members of the public.

Ethics and Dissemination

The design of a pathway and underpinning patient preference will assist in understanding factors which might influence technology implementation for clinical care. This study requires ethical approval for phases 2, 3 and 4. Approvals have been received for phase 2 (Approval received 4th December 2016 from the South Central-Oxford C Research Ethics Committee [IRAS ID: 255172, REC Reference 18/SC/0663) and 3 (Approval received 18th October 2019 from the London-Hampstead Research Ethics Committee [IRAS ID: 248064, REC Reference 19/LO/1586]) and will be sought for phase 4. All participants will provide informed written consent prior to being enrolled onto the study.

For peer review only - http://bmjopen.bmj.com/site/about/guidelines.xhtml

A manuscript will be written for publication for each phase and submitted to National and International Conferences. In addition, lay summary results will be developed and made available for patients and members of the public. All results will be published in open access peer reviewed journals. Links to research outputs will be made available on the CONNECT Project website available at www.theconnectproject.info.

Acknowledgements

The authors thank members of the CONNECT Project patient and public involvement steering group for their invaluable contributions to the study design. The authors also thank Rachel Dalton, John Doyle, Anju Jaggi, Iva Hauptmannova, Rosalind Moss, John Skinner and colleagues within the Therapies Directorate and Research and Innovation Centre at the Royal National Orthopaedic Hospital for their ongoing support

Contributors

AG wrote the paper and conceived the project with the support of CRM, JJ and MS. CRM contributed knowledge on systematic reviews and qualitative research. JJ and EM contributed knowledge on the DCE. CRM, JJ, MS and EM edited and critically revised the paper. All authors have read and approved the manuscript.

Funding Statement

Anthony Gilbert is funded by a National Institute for Health Research (NIHR), Clinical Doctoral Research Fellowship for this research project (ICA-CDRF-2017-03-025).

Disclaimer

This paper presents independent research funded by the National Institute for Health Research (NIHR). The views expressed are those of the authors and not necessarily those of the NHS, the NIHR or the Department of Health and Social Care.

Competing Interests

Nil.

Patient Consent for Publication

Not required.

For peer review only

For peer review only - http://bmjopen.bmj.com/site/about/guidelines.xhtml

References

1. Mahendradhata Y, Trisnantoro L, Listyadewi S, et al. The Republic of Indonesia health system review: Health Systems in Transition, 2017:https://apps.who.int/iris/bitstream/handle/10665/254716/9789290225164-eng.pdf;sequence=1.

2. Tan X, Liu X, Shao H. Healthy China 2030: A Vision for Health Care. *Value in Health Regional Issues* 2017;12:112-14. doi: 10.1016/j.vhri.2017.04.001

3. Department of Health. National Strategic Framework for Rural and Remote Health. 2016. https://www1.health.gov.au/internet/main/publishing.nsf/Content/A76BD33A5D7A6897CA257F9B00095DA3/$File/National%20Strategic%20Framework%20for%20Rural%20and%20Remote%20Health.pdf (Accessed 3rd October 2019)

4. NHS. The NHS Long Term Plan. 2019. https://www.longtermplan.nhs.uk/wp-content/uploads/2019/01/nhs-long-term-plan-june-2019.pdf (Accessed 3rd October 2019).

5. Department of Health and Social Care. NHS Mandate 2018 to 2019. 2018. https://assets.publishing.service.gov.uk/government/uploads/system/uploads/attachment_data/file/803111/revised-mandate-to-nhs-england-2018-to-2019.pdf (Accessed 18th August 2019).

6. World Health Organisation. Musculoskeletal conditions fact sheet. 2019. https://www.who.int/news-room/fact-sheets/detail/musculoskeletal-conditions (Accessed 3rd October 2019).

7. Felson DT, Naimark A, Anderson J, et al. The prevalence of knee osteoarthritis in the elderly. the framingham osteoarthritis study. *Arthritis & Rheumatism* 1987;30(8):914-18. doi: 10.1002/art.1780300811

8. Deshpande BR, Katz JN, Solomon DH, et al. Number of Persons With Symptomatic Knee Osteoarthritis in the US: Impact of Race and Ethnicity, Age, Sex, and Obesity. *Arthritis Care and Research* 2016;68(12):1743-50. doi: 10.1002/acr.22897

9. Wallace P, Haines A, Harrison R, et al. Joint teleconsultations (virtual outreach) versus standard outpatient appointments for patients referred by their general practitioner for a specialist opinion: a randomised trial. *The Lancet* 2002;359(9322):1961-68. doi: 10.1016/S0140-6736(02)08828-1

10. Salisbury C, Montgomery A, Hollinghurst S, et al. Effectiveness of PhysioDirect telephone assessment and advice services for patients with musculoskeletal problems: pragmatic randomised controlled trial. *BMJ: British Medical Journal* 2013;346(7893):14. doi: 10.1136/bmj.f43

11. Armfield NR, Bradford M, Bradford NK. The clinical use of Skype—For which patients, with which problems and in which settings? A snapshot review of the literature. *International Journal of Medical Informatics* 2015;84(10):737-42. doi: 10.1016/j.ijmedinf.2015.06.006

12. Greenhalgh T, Vijayaraghavan S, Wherton J, et al. Virtual online consultations: advantages and limitations (VOCAL) study. *BMJ Open.* 2016. doi: 10.1136/bmjopen-2015-009388

13. Gilbert AW, Jaggi A, May CR. What is the acceptability of real time 1:1 videoconferencing between clinicians and patients for a follow-up consultation for multi-directional shoulder instability? *Shoulder & Elbow* 2019;11(1):53-59. doi: 10.1177/1758573218796815

For peer review only - http://bmjopen.bmj.com/site/about/guidelines.xhtml

14. May C. A rational model for assessing and evaluating complex interventions in health care. *BMC Health Services Research*. 2006;6. doi: 10.1186/1472-6963-6-86

15. Carl M, Tracy F. Implementing, Embedding, and Integrating Practices: An Outline of Normalization Process Theory. *Sociology* 2009;43(3):535.

16. May C. Towards a general theory of implementation. *Implementation Science* 2013;8(1) doi: 10.1186/1748-5908-8-18

17. May CR, Johnson M, Finch T. Implementation, context and complexity. *Implementation Science.* 2016;11. doi: 10.1186/s13012-016-0506-3

18. Gallacher K, Mair FS, May CR, et al. Understanding patients' experiences of treatment burden in chronic heart failure using normalization process theory. *Annals of Family Medicine* 2011;9(3):235-43. doi: 10.1370/afm.1249

19. Gallacher K, Jani B, Morrison D, et al. Qualitative systematic reviews of treatment burden in stroke, heart failure and diabetes - Methodological challenges and solutions. *BMC Medical Research Methodology* 2013(1):10. doi: 10.1186/1471-2288-13-10

20. Lippiett KA, Richardson A, Myall M, et al. Patients and informal caregivers' experiences of burden of treatment in lung cancer and chronic obstructive pulmonary disease (COPD): a systematic review and synthesis of qualitative research. *BMJ Open* 2019;9(2):e020515-e15. doi: 10.1136/bmjopen-2017-020515

21. May CR, Eton DT, Boehmer K, et al. Rethinking the patient: using Burden of Treatment Theory to understand the changing dynamics of illness. *BMC Health Services Research* 2014;14:281-81. doi: 10.1186/1472-6963-14-281

22. Carl M, Victor MM, Frances SM. We need minimally disruptive medicine. *BMJ: British Medical Journal* 2009;339(7719):485. doi: 10.1136/bmj.b2803

23. Shippee ND, Allen SV, Leppin AL, et al. Attaining minimally disruptive medicine: Context, challenges and a roadmap for implementation. *Journal of the Royal College of Physicians of Edinburgh* 2015;45(2):118-22. doi: 10.4997/JRCPE.2015.206

24. Tavory I, Timmermans S. Abductive Analysis: Theorizing Qualitative Research: Chicago and London: University of Chicago Press 2014.

25. Gilbrt AW, Jaggi A, May CR, et al. What is the work of being a patient when using communication technology to access healthcare in an orthopaedic setting? A systematic review and meta-synthesis of qualitative studies. PROSPERO CRD42018100896. 2018. http://www.crd.york.ac.uk/PROSPERO/display_record.php?ID=CRD42018100896 (Accessed 3rd October 2019)

26. Critical Appraisal Skills Programme. CASP Qualitative Checklist. 2018 https://casp-uk.net/wp-content/uploads/2018/01/CASP-Qualitative-Checklist-2018.pdf (accessed 18th June 2019).

27. Ryan M, Gerard K, Amaya-Amaya Me. Using Discrete Choice Experiments to Value Health and Health Care: Economics of Non-Market Goods and Resources series, vol. 11. Dordrecht: Springer 2008.

For peer review only - http://bmjopen.bmj.com/site/about/guidelines.xhtml

Figure 1: Overview of the CONNECT PROJECT

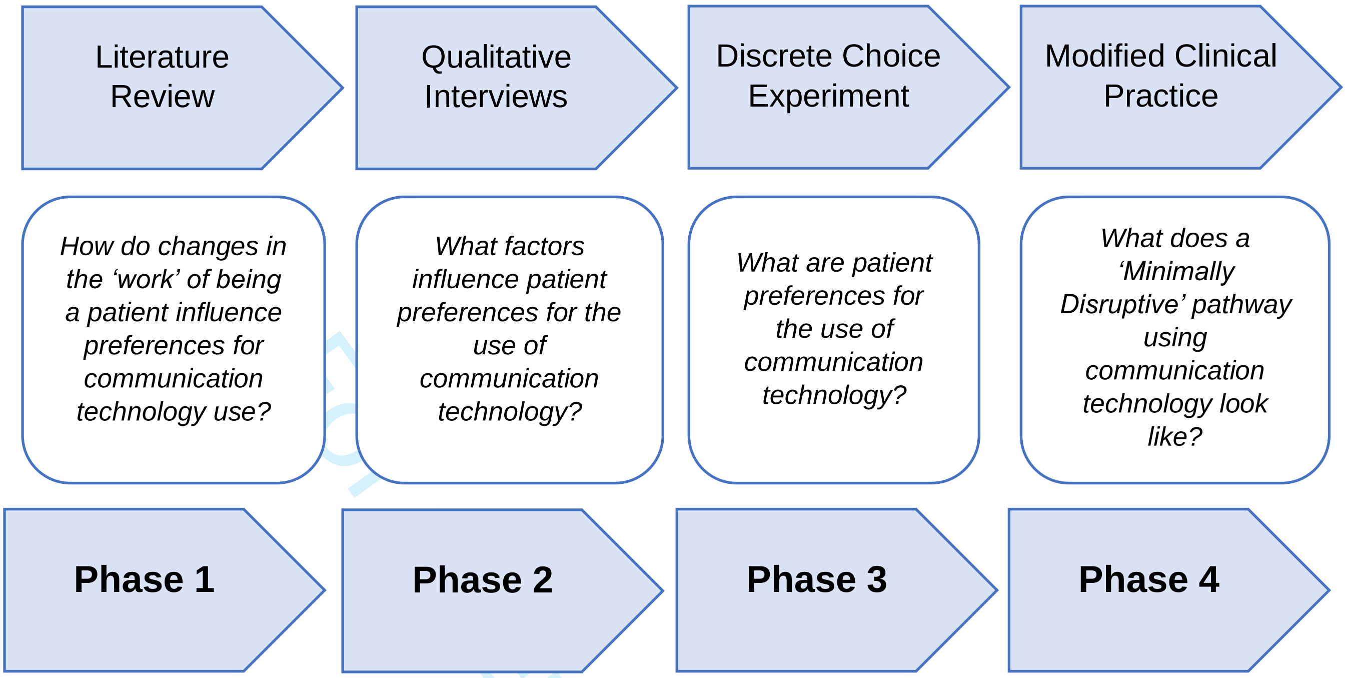

BMJ Open

Protocol for the CONNECT Project: a mixed methods study investigating patient preferences for communication technology use in orthopaedic rehabilitation consultations

Journal:	*BMJ Open*
Manuscript ID	bmjopen-2019-035210.R1
Article Type:	Protocol
Date Submitted by the Author:	08-Nov-2019
Complete List of Authors:	Gilbert, Anthony; Royal National Orthopaedic Hospital Stanmore, Therapies Department; University of Southampton, Faculty of Health Sciences<br>Jones, Jeremy; University of Southampton, Faculty of Health Sciences<br>Stokes, Maria; University of Southampton, Faculty of Health Sciences<br>Mentzakis, Emmanouil; University of Southapton, Faculty of Economic, Social and Political Science<br>May, Carl; London School of Hygiene and Tropical Medicine Faculty of Epidemiology and Population Health
**Primary Subject Heading**:	Health services research
Secondary Subject Heading:	Health economics, Qualitative research
Keywords:	Adult orthopaedics < ORTHOPAEDIC & TRAUMA SURGERY, Musculoskeletal disorders < ORTHOPAEDIC & TRAUMA SURGERY, Telemedicine < BIOTECHNOLOGY & BIOINFORMATICS

SCHOLARONE™
Manuscripts

Protocol for the CONNECT Project: a mixed methods study investigating patient preferences for communication technology use in orthopaedic rehabilitation consultations

Authors

1. Anthony Gilbert[1, 2]
2. Jeremy Jones[2]
3. Maria Stokes[2]
4. Emmanouil Mentzakis[3]
5. Carl R May[4]

Author Affiliations

1. Therapies Department, Royal National Orthopaedic Hospital, Stanmore, UK

2. School of Health Sciences, Faculty of Environment and Life Sciences, University of Southampton, Southampton, UK

3. Faculty of Economic, Social and Political Science, University of Southampton, Southampton, UK

4. Faculty of Public Health and Policy, London School of Hygiene and Tropical Medicine, London, UK

Corresponding author: Anthony Gilbert anthony.gilbert@nhs.net

For peer review only

For peer review only - http://bmjopen.bmj.com/site/about/guidelines.xhtml

Abstract

Introduction

Technology has been placed at the centre of global health policy and has been cited as having the potential to increase efficiency and remove geographical boundaries for patients to access care. Communication technology may support patients with orthopaedic problems which is one of the leading causes of disability worldwide. There are several examples of technology being used in clinical research although uptake in practice remains low. An understanding of patient preferences will support the design of a communication technology supported treatment pathway for patients undergoing orthopaedic rehabilitation.

Methods and Analysis

This mixed methods project will be conducted in four phases. In phase 1 a systematic review of qualitative studies reporting communication technology use for orthopaedic rehabilitation will be conducted to devise a taxonomy of tasks patients face when using these technologies to access their care. In phase 2 qualitative interviews will investigate how the work of being a patient changes during face-to-face and communication technology consultations and how these changes influence preference. In Phase 3 a Discrete Choice Experiment will investigate the factors that influence preferences for the use of communication technology for orthopaedic rehabilitation consultations. Phase 4 will be a practical application of these results. We will design a 'minimally disruptive' communication technology supported pathway for patients undergoing orthopaedic rehabilitation.

Ethics and Dissemination

The design of a pathway and underpinning patient preference will assist in understanding factors which might influence technology implementation for clinical care. This study requires ethical approval for phases 2, 3 and 4. Approvals have been received for phase 2 (Approval received 4th December 2016 from the South Central-Oxford C Research Ethics Committee [IRAS ID: 255172, REC Reference 18/SC/0663) and 3 (Approval received 18th October 2019 from the London-Hampstead Research Ethics Committee [IRAS ID: 248064, REC Reference 19/LO/1586]) and will be sought for phase 4. All participants will provide informed written consent prior to being enrolled onto the study.

Registration

PROSPERO registration number: ID=CRD42018100896.

For peer review only - http://bmjopen.bmj.com/site/about/guidelines.xhtml

Strengths and Limitations of this Study

- A taxonomy of patient 'work' and characterization of patient preferences when using communication technology will assist in understanding implementation processes.
- This combination of sociological and economic research methods is novel: there are very few studies of patient preferences in telemedicine research.
- The design of a new consultation pathway, underpinned by patient preferences, may enhance the prospects of successful implementation in practice.
- This research is being conducted across two sites and may not be representative of the NHS nationwide.

For peer review only

For peer review only - http://bmjopen.bmj.com/site/about/guidelines.xhtml

Introduction

Technology has been placed at the center of global healthcare policy. Technology has been cited as having the potential to improve the effectiveness of healthcare systems through efficiency gain strategies[1] and healthcare reform[2]. Technology may overcome geographical boundaries[3] with one example, from the Republic of Indonesia Health System Review[1], stating 'A telemedicine network would enable patients in remote areas to have access to reliable medical consultations, and at the same time health professionals in remote areas can also be supported through the use of telemedicine technology'. In the United Kingdom, as outlined in the NHS Long Term Plan[4], digital-first primary care will become a new option for every patient intending to provide fast access to convenient primary care with 95% of GP patients to be offered e-consultation and other digital services in 2019[5].

Musculoskeletal disease is the second largest cause of disability worldwide[6]. It is widely accepted that the presence of osteoarthritis (OA) increases with age[7] although more than half of people with symptomatic OA are younger than 65[8]. It is likely that many of these younger people will live for another 2-3 decades and require ongoing support and management that requires visits to healthcare practitioners. Communication technology, the use of technology to support the communication from a distance, is a digital innovation that can support patients to attend appointments.

There are several examples of communication technology to support the management of musculoskeletal disorders in the literature. The Virtual Outreach Project[9] compared joint teleconsultations between hospital specialists, General Practitioners and their patients in the United Kingdom and found the Virtual Outreach group to have significant increases in satisfaction compared to the face-to-face group. PhysioDirect[10] telephone assessment was found to be as effective as face-to-face care for patients with musculoskeletal disorders accessing their care via phone. Skype, a free-to-access videoconferencing software, has been used across a range of clinical specialities[11]. Greenhalgh's VOCAL study[12] found video outpatient consultations to be safe, effective and convenient in appropriate situations. Our previous research found the use of Skype videoconferencing for patients with shoulder instability to be acceptable for half of the patients[13]. In our study, there were several factors that influenced patient's choices between face-to-face and Skype consultations. We believe that further research on this area may assist with implementation of communication technology in clinical practice.

The process of implementing a new intervention (such as the introduction of communication technology in healthcare) has been demonstrated to be dependent on how the intervention is operationalised by its users[14], the "work" people do when they implement a new intervention[15], the mobilisation of resources over time[16] across different settings[17]. Normalisation Process Theory (NPT) frames implementation processes through its focus on the things people *do* when they implement a new intervention in practice and provides the theoretical underpinning of phase 1.

For peer review only - http://bmjopen.bmj.com/site/about/guidelines.xhtml

NPT has been used to determine the components of patient "work" in chronic heart failure[18], stroke and diabetes[19] and Chronic Obstructive Pulmonary Disease and lung cancer[20]. Patient work in heart failure includes the work of developing an understanding of treatments, interacting with others to organise care, attending appointments, taking medications, enacting lifestyle measures and appraising treatments. Burden of treatment theory (BOT)[21] explains how the capacity for action interacts with the work that stems from healthcare. We are particularly interested in BOT across different situation of consultation and BOT provides the theoretical underpinning of phase 2.

Minimally Disruptive Medicine (MDM)[22] is an approach to healthcare that seeks to reduce the workload for the patient and caregiver. MDM seeks to advance patient goals for healthcare using effective care programmes designed and implemented in a manner that minimises the negative impact the care programme imposes on their lives[23]. A 'minimally disruptive' orthopaedic rehabilitation consultation is a consultation that:

- has minimal negative imposition on the patient's life
- offers a reduce workload for the patient
- ensures healthcare professionals and care are accessible to the patient

The CONNECT Project utilises the aforementioned theories to understand:

(i) the workload of being a patient when using communication technology (using NPT);
(ii) how the situational nature of a communication technology and face-to-face consultation influence burden of treatment (using BOT) and patient preferences;
(iii) patient preference in relation face to face and communication technology consultations;
(iv) what a 'minimally disruptive' orthopaedic rehabilitation consultation looks like in practice (MDM).

Population

Adults ≥ 18 years of age with orthopaedic conditions.

Philosophical Underpinnings

This study is set within the abduction paradigm[24]. Abduction is the production of a hypothesis based on surprising evidence and, when following this approach, researchers seek to choose the 'best' amongst many alternatives. Abduction sits in the philosophical tradition of pragmatism, an ideology that supports the notion that a proposition is true when it works satisfactorily. Within the context of this research, one can make assumptions that 'certain' patients may prefer virtual appointments to face-to-face appointments (or vice versa).

We hypothesise that certain patients may indicate they prefer virtual appointments to face-to-face appointments (or vice versa). Large scale data collection in phase 3 will support theorisation of preference in this study group. The purpose of the research is to develop satisfactory propositions, based on these data, to explain patient preferences and to design a minimally disruptive pathway based on these propositions.

Overall Aim

To understand the patient preferences for the use of communication technology in orthopaedic rehabilitation consultations and design a 'minimally disruptive' consultation pathway based on these preferences.

For peer review only - http://bmjopen.bmj.com/site/about/guidelines.xhtml

Health Condition

Patients with orthopaedic problems.

Methods & Analysis

An overview of the four phases of the CONNECT Project is shown in Figure 1.

Phase 1: Systematic Review

We are interested in how the 'work' of being a patient influences preference. To the authors knowledge, no research has yet considered how the work of being a patient influences preference for communication technology consultations. The purpose of phase 1 is to develop a taxonomy of tasks required of patients using communication technology. We will then consider how factors relating to these tasks influence the comparative evaluation patients are faced when offered the choice of a communication technology or a face-to-face consultation for orthopaedic rehabilitation. This systematic review will be conducted using the PRISMA approach in order to answer the research question: *How do changes in the 'work' of being a patient when using communication technology influence patient preferences?* The protocol for this review was registered on the International prospective register of systematic reviews (PROSPERO registration number: ID=CRD42018100896)[25]. The PRISMA Protocol (Prisma P) is demonstrated in Supplementary File 1.

MEDLINE, AMED, CINAHL, PsychINFO and SCOPUS will be searched from inception. The full search strategy, with search terms for each database, is available as a supplementary file (Supplementary File 2). Following the search, articles will be screened independently by two authors to identify full text studies to be included in the review. A third author will be available to discuss any discrepancies.

Studies will be eligible for inclusion providing they meet the criteria for inclusion shown in Table 1. Relevant studies will be firstly screened by their title and then by their abstract. Remaining texts will then read in full with all texts retained after this point for qualitative synthesis. Risk of bias will be screened using the CASP tool for qualitative studies[26]. A discussion will be held between the authors to decide whether included studies are of sufficient quality to include in the review. A third author will be available to discuss any discrepancies. Reasons for exclusion will be listed.

For peer review only

Table 1: Eligibility Criteria of Studies

Inclusion:	Exclusion:
• Full text academic papers.<br>Participants:<br>• Patients with an orthopaedic / musculoskeletal problem<br>Intervention:<br>• Studies reporting patients accessing physical assessment / rehabilitation using communication technology (e.g. telephone, videoconferencing) in an orthopaedic / musculoskeletal setting.<br>Outcome:<br>• Qualitative studies or studies with a qualitative component that focuses on the patient viewpoint of accessing communication technology.	• Conference abstracts<br>• Participants without an orthopaedic / musculoskeletal complaint<br>• Quantitative studies<br>• Studies not reporting patient viewpoints

Full texts will be uploaded to QSR NVIVO Software (QSR International Pty Ltd. Version 12, 2018). NVIVO will be used to collect and organise data from the results, discussion and conclusion sections of each paper. Data will be collected by one author (AG). For the purpose of data collection, the introduction and methods will be disregarded. The following process will then be followed:

1. Each sentence from the results, discussion and conclusion sections from the papers will be extracted and coded in NVIVO on a line by line basis. The codes will be attributed to each sentence based on their content.
2. An Abductive Analysis[24] will then be conducted and will take three forms:
    a. Firstly, a thematic analysis of codes. This will enable authors to familiarise themselves with the content of the papers.
    b. The following will be considered: *what is the work of being a patient when using communication technology?* Codes will then be organised into groups of codes depicting the *type* of work required of patients when using communication technology to access healthcare in order to develop a taxonomy of the types of work.
    c. We will consider the question: *how might the work of being a patient when using communication technology influence patient preference?*
3. Data will be mapped out in the form of a model to demonstrate how, based on the included papers, the change in the 'work' of being a patient might influence preference for communication technology.

Phase 2: Qualitative Interviews

Ethical Approval received 4th December 2016 from the South Central-Oxford C Research Ethics Committee (IRAS ID: 255172, REC Reference 18/SC/0663).

The aim of phase 2 is to explore how the use of communication technology changes the experience for patients receiving physiotherapy and occupational therapy for orthopaedic problems. This study will be conducted at one hospital. The results from phase 1 will frame the initial enquiry and

For peer review only - http://bmjopen.bmj.com/site/about/guidelines.xhtml

interview schedule for phase 2. Questions relating to Burden of Treatment Theory[21] will explore the potential impact and workload changes for patients with the use of these technologies. The research question for phase 2 is: *How does communication technology use affect patient experience?* A focus on the circumstances in which patients would prefer to use communication technology will be used to inform the design of a Discrete Choice Experiment (DCE) for phase 3 of the CONNECT Project. These viewpoints (Phase 2) and the DCE (Phase 3) will inform the design of a modified clinical pathway (Phase 4).

This study will use qualitative methodology to gain rich data regarding patient and clinicians' opinions. Qualitative methods have been chosen to explore the underlying reasons behind these opinions. Semi structured interviews have been chosen to provide a loose guide and enable the researcher to explore pertinent themes relating to the research aims and objectives without the rigidity of a survey. The research paper reporting the results of phase 2 will be reported using the SRQR Checklist (the checklist for this protocol paper is available in Supplementary File 3).

The study will be conducted at one hospital site (a tertiary orthopaedic hospital). Participants will be recruited from the occupational and physiotherapy department of the hospital site. This study will aim to recruit 20 patients (5 males, 5 females under the age of 49; 5 males, 5 females aged 50 and over) and 20 clinicians comprising of physiotherapists and occupational therapists (at least 8 occupational therapists). This number has been selected to allow for a broad range of views within the scope and resources of a sub-study within a PhD project. Patients are eligible for inclusion if they meet the inclusion criteria shown in Table 2.

<u>Table 2: Inclusion / Exclusion criteria for Phase 2</u>

Inclusion:	Exclusion:
• Patients, over the age of 18 years, attending the hospital site for Physiotherapy or Occupational Therapy<br>• Patients who have experience of orthopaedic / musculoskeletal condition<br>• Patients who are able to provide informed written consent to enter into the study<br>• Patients able to understand and speak English or a language covered by the hospitals Interpreter service	• Patients without the capacity to consent<br>• Patients suffering from disorders other than orthopaedic as the primary cause (e.g. neurological or oncology disorders)

Participants who are eligible to enrol will be given a participant information sheet. All participants will have at least 24 hours to consider their participation and ask questions before being asked to provide informed, written consent. Upon receipt of consent the participant will be recruited into the study. All participants will receive a copy of the consent form and a copy will also be saved in the project file. The lead researcher (AG) is a practicing physiotherapist at the hospital site. Patients will not be eligible for inclusion if they have previously, or are currently, been treated by AG. At a mutually convenient time, the participants will be interviewed by AG, either face-to-face or via video call using Skype or Zoom software. Interviews will be conducted using an interview guide developed upon completion of phase 1. All interviews will be audio recorded. All recordings will be linked anonymised using a unique study identifier, stored on an NHS Password encrypted computer and be sent off to an external company to be transcribed verbatim.

For peer review only - http://bmjopen.bmj.com/site/about/guidelines.xhtml

Upon receipt of the transcriptions, copies will be posted to all participants with an enclosed stamped addressed envelope. Participants will be given two weeks to review the transcriptions for factual accuracy and given the opportunity to add any additional comments. Transcripts will not be amended if the participant does not return them. At this stage no other input will be required from research participants.

Upon receipt of amended transcripts or confirmation that no changes are required, transcripts will be uploaded into NVIVO software for organisation of data. Each sentence from the included sections will be coded in NVIVO on a line by line basis. The codes will be labelled using a description of the content of the respective sentence. Data analysis will take three forms: firstly, a thematic analysis of codes. This will enable researchers to familiarise themselves with the content of the interviews. For the second iteration of coding the following will be considered: *what is the work of being a patient when using communication technology?* Codes will then be organised into groups of codes depicting the *type* of work required of patients when using communication technology to access healthcare in order to develop a taxonomy of the types of work. The coding will be completed in a way that looks to extend the model in phase 1. Throughout this process, we will consider the question: *how might the work of being a patient when using communication technology influence patient preference?* Specific data to support the design and development of future components of the CONNECT Project (namely phase 3) will be organised separately.

Phase 3: Discrete Choice Experiment

Ethical Approval received 18th October 2019 from the London-Hampstead Research Ethics Committee (IRAS ID: 248064, REC Reference 19/LO/1586).

The aim of phase 3 is to understand the factors that influence patient preference when presented with the choice between a face-to-face and communication technology consultation for orthopaedic rehabilitation. The research question for phase 3 is *what factors influence preferences for patients undergoing orthopaedic rehabilitation who are offered a face-to-face or communication technology consultation?*

The results from phase 1 and phase 2 will inform the design of the DCE. It is not clear at this stage what the attributes and individual levels will be. However, they are likely to include travel time and cost, perceived ease of use of equipment to engage in the consultation, raining requirements, conduct and content of the consultation and the number of engagements with clinicians during any given pathway.

A D-efficient design will be created in NGene software (Choice Metrics) where attribute non-linearity will be allowed (i.e. levels of specific attributes allowed to have non-linear effects). To reduce cognitive burden on participants the maximum number of choice sets will be limited to 12 and blocking will be utilized if required (i.e. blocking implies orthogonally splitting the number choice sets into two or more groups which are then presented to different individuals).

This study will be conducted across two hospital sites (a tertiary orthopaedic hospital and a secondary care orthopaedic hospital). Participants will be recruited from the occupational and physiotherapy department of the hospital site. This study will aim to recruit at least 200 patients per site. Patients are eligible for inclusion if they meet the inclusion criteria shown in Table 3.

<u>Table 3: Inclusion / Exclusion criteria for Phase 3</u>

Inclusion:	Exclusion:

For peer review only - http://bmjopen.bmj.com/site/about/guidelines.xhtml

• Patients, over the age of 18 years, attending either hospital site for Physiotherapy or Occupational Therapy<br>• Patients who have experience of orthopaedic / musculoskeletal condition<br>• Patients who are able to provide informed written consent to enter into the study<br>• Patients able to understand and speak English or a language covered by the hospitals Interpreter service	• Patients without the capacity to consent<br>• Patients suffering from disorders other than orthopaedic as the primary cause (e.g. neurological or oncology disorders)

It is anticipated that around 200 participants per site will be recruited but precise numbers will be dependent on a power analysis once the number of questions and blocks has been ascertained. Participants who are eligible to enrol will be given a participant information sheet. All participants will be asked to provide informed, written consent at that time. Upon receipt of consent the participant will be recruited into the study. All participants will receive a copy of the consent form and a copy will also be saved in the project file.

The DCE questionnaire will be designed using online questionnaire software (Survey Monkey). The DCE will be administered in the choice of 2 forms - paper or electronically using a tablet computer – and patients will be offered the choice of completing at the study site or at home within 24 hours. All participants will be provided with an envelope to return the completed DCE questionnaire. Data from paper questionnaires will be manually entered by the researcher. Online SurveyMonkey questionnaires automatically exports data into Microsoft Excel. The initial questionnaire will be piloted on approximately 10 patients. This will undergo repeat piloting on further iterations of the DCE until the final design is established.

Initial reporting will provide descriptive data for demographic variables and observed choices (virtual vs face-to-face) by choice set. Given the binary set-up of the experiment, analysis will proceed with conditional logit and random parameter binary models[27]. Attribute levels will enter as covariates to explain individual choices, while individual specific characteristics will either enter as interactions with attribute or directly, depending on the estimation model used. Following standard literature, unobserved heterogeneity, if present, will be explored through a random coefficient model. Trade-offs and marginal rates of substitution between attribute level will be calculated, while willingness-to-pay values will also be computed if cost is present in the final list of attributes for the experiment. The resulting factors that influence preferences will be used to further develop the model of patient preference from phases 1 & 2.

Phase 4: Pathway Design

The aim of phase 4 is to design a model of care based on the results from phase 1-3. The research question for this phase is *what does a minimally disruptive consultation look like in orthopaedic rehabilitation?* A theoretical model of care developed during the results of phases 1-3 will be applied practically to orthopaedic rehabilitation. The model of rehabilitation will be designed and piloted on a small number of patients (approximately 10) and their clinicians to understand the impact of the new consultation format. A small study of acceptability will be conducted with a view to inform the further development of the model of care and to gain insight into the issues that might influence further upscale and transportability of the model of care in other settings. Ethical and Health Research Authority approval will be sought prior to commencing this phase.

For peer review only - http://bmjopen.bmj.com/site/about/guidelines.xhtml

Potential benefits to patients and to the NHS

Previous studies into the introduction of e-health technologies have used top down models in which the methodologies and interventions have been decided by investigators without a complete understanding of patient preferences. These studies, although pointing to the value of e-health technologies, have not always led to routine uptake in clinical practice. The CONNECT Project investigates the role of patient preferences in normalization processes, and it is postulated that the knowledge of such patient preferences is more likely to lead to successful e-health implementation. This project will focus on orthopaedic rehabilitation appointments, but it will have much wider implications for the introduction of e-health technology to other spheres of medicine. There is the potential to both provide a better patient service and to effect cost savings to society and the healthcare system.

Patient and Public Involvement

The CONNECT Project Patient and Public Involvement steering group (PPISG) has been set up to provide guidance on the conduct of the research (details available from www.theconnectproject.info). The first meeting of the PPISG was held in August 2016 prior to the submission of the research to the NIHR in May 2017. A discussion was held about the overall research aims which supported the identification of the research questions. The PPISG has supported the design of the overall research plan and will continue to be involved during the development and refinement of each phase prior to the completion of each study protocol. The participant information and consent forms and the discrete choice experiement questionnaire for phase 3 has been reviewed by the PPISG. In addition, the PPISG will support the development of the lay-summary outputs to be disseminated to patients and members of the public.

Ethics and Dissemination

The design of a pathway and underpinning patient preference will assist in understanding factors which might influence technology implementation for clinical care. This study requires ethical approval for phases 2, 3 and 4. Approvals have been received for phase 2 (Approval received 4th December 2016 from the South Central-Oxford C Research Ethics Committee [IRAS ID: 255172, REC Reference 18/SC/0663) and 3 (Approval received 18th October 2019 from the London-Hampstead Research Ethics Committee [IRAS ID: 248064, REC Reference 19/LO/1586]) and will be sought for phase 4. All participants will provide informed written consent prior to being enrolled onto the study.

A manuscript will be written for publication for each phase and submitted to National and International Conferences. In addition, lay summary results will be developed and made available for patients and members of the public. All results will be published in open access peer reviewed journals. Links to research outputs will be made available on the CONNECT Project website available at www.theconnectproject.info.

Acknowledgements

The authors thank members of the CONNECT Project patient and public involvement steering group for their invaluable contributions to the study design. The authors also thank Rachel Dalton, John Doyle, Anju Jaggi, Iva Hauptmannova, Rosalind Moss, John Skinner and colleagues within the Therapies Directorate and Research and Innovation Centre at the Royal National Orthopaedic Hospital for their ongoing support

For peer review only - http://bmjopen.bmj.com/site/about/guidelines.xhtml

Contributors

AG wrote the paper and conceived the project with the support of CRM, JJ and MS. CRM contributed knowledge on systematic reviews and qualitative research. JJ and EM contributed knowledge on the DCE. CRM, JJ, MS and EM edited and critically revised the paper. All authors have read and approved the manuscript. AG is the guarantor of the manuscript.

Funding Statement

Anthony Gilbert is funded by a National Institute for Health Research (NIHR), Clinical Doctoral Research Fellowship for this research project (ICA-CDRF-2017-03-025).

Disclaimer

This paper presents independent research funded by the National Institute for Health Research (NIHR). The views expressed are those of the authors and not necessarily those of the NHS, the NIHR or the Department of Health and Social Care.

Competing Interests

Nil.

Patient Consent for Publication

Not required.

Figure 1 – Overview of the CONNECT Project.

For peer review only - http://bmjopen.bmj.com/site/about/guidelines.xhtml

References

1. Mahendradhata Y, Trisnantoro L, Listyadewi S, et al. The Republic of Indonesia health system review: Health Systems in Transition, 2017:https://apps.who.int/iris/bitstream/handle/10665/254716/9789290225164-eng.pdf;sequence=1.

2. Tan X, Liu X, Shao H. Healthy China 2030: A Vision for Health Care. *Value in Health Regional Issues* 2017;12:112-14. doi: 10.1016/j.vhri.2017.04.001

3. Department of Health. National Strategic Framework for Rural and Remote Health. 2016. https://www1.health.gov.au/internet/main/publishing.nsf/Content/A76BD33A5D7A6897CA257F9B00095DA3/$File/National%20Strategic%20Framework%20for%20Rural%20and%20Remote%20Health.pdf (Accessed 3rd October 2019)

4. NHS. The NHS Long Term Plan. 2019. https://www.longtermplan.nhs.uk/wp-content/uploads/2019/01/nhs-long-term-plan-june-2019.pdf (Accessed 3rd October 2019).

5. Department of Health and Social Care. NHS Mandate 2018 to 2019. 2018. https://assets.publishing.service.gov.uk/government/uploads/system/uploads/attachment_data/file/803111/revised-mandate-to-nhs-england-2018-to-2019.pdf (Accessed 18th August 2019).

6. World Health Organisation. Musculoskeletal conditions fact sheet. 2019. https://www.who.int/news-room/fact-sheets/detail/musculoskeletal-conditions (Accessed 3rd October 2019).

7. Felson DT, Naimark A, Anderson J, et al. The prevalence of knee osteoarthritis in the elderly. the framingham osteoarthritis study. *Arthritis & Rheumatism* 1987;30(8):914-18. doi: 10.1002/art.1780300811

8. Deshpande BR, Katz JN, Solomon DH, et al. Number of Persons With Symptomatic Knee Osteoarthritis in the US: Impact of Race and Ethnicity, Age, Sex, and Obesity. *Arthritis Care and Research* 2016;68(12):1743-50. doi: 10.1002/acr.22897

9. Wallace P, Haines A, Harrison R, et al. Joint teleconsultations (virtual outreach) versus standard outpatient appointments for patients referred by their general practitioner for a specialist opinion: a randomised trial. *The Lancet* 2002;359(9322):1961-68. doi: 10.1016/S0140-6736(02)08828-1

10. Salisbury C, Montgomery A, Hollinghurst S, et al. Effectiveness of PhysioDirect telephone assessment and advice services for patients with musculoskeletal problems: pragmatic randomised controlled trial. *BMJ: British Medical Journal* 2013;346(7893):14. doi: 10.1136/bmj.f43

11. Armfield NR, Bradford M, Bradford NK. The clinical use of Skype—For which patients, with which problems and in which settings? A snapshot review of the literature. *International Journal of Medical Informatics* 2015;84(10):737-42. doi: 10.1016/j.ijmedinf.2015.06.006

12. Greenhalgh T, Vijayaraghavan S, Wherton J, et al. Virtual online consultations: advantages and limitations (VOCAL) study. *BMJ Open.* 2016. doi: 10.1136/bmjopen-2015-009388

13. Gilbert AW, Jaggi A, May CR. What is the acceptability of real time 1:1 videoconferencing between clinicians and patients for a follow-up consultation for multi-directional shoulder instability? *Shoulder & Elbow* 2019;11(1):53-59. doi: 10.1177/1758573218796815

For peer review only - http://bmjopen.bmj.com/site/about/guidelines.xhtml

14. May C. A rational model for assessing and evaluating complex interventions in health care. *BMC Health Services Research*. 2006;6. doi: 10.1186/1472-6963-6-86

15. Carl M, Tracy F. Implementing, Embedding, and Integrating Practices: An Outline of Normalization Process Theory. *Sociology* 2009;43(3):535.

16. May C. Towards a general theory of implementation. *Implementation Science* 2013;8(1) doi: 10.1186/1748-5908-8-18

17. May CR, Johnson M, Finch T. Implementation, context and complexity. *Implementation Science.* 2016;11. doi: 10.1186/s13012-016-0506-3

18. Gallacher K, Mair FS, May CR, et al. Understanding patients' experiences of treatment burden in chronic heart failure using normalization process theory. *Annals of Family Medicine* 2011;9(3):235-43. doi: 10.1370/afm.1249

19. Gallacher K, Jani B, Morrison D, et al. Qualitative systematic reviews of treatment burden in stroke, heart failure and diabetes - Methodological challenges and solutions. *BMC Medical Research Methodology* 2013(1):10. doi: 10.1186/1471-2288-13-10

20. Lippiett KA, Richardson A, Myall M, et al. Patients and informal caregivers' experiences of burden of treatment in lung cancer and chronic obstructive pulmonary disease (COPD): a systematic review and synthesis of qualitative research. *BMJ Open* 2019;9(2):e020515-e15. doi: 10.1136/bmjopen-2017-020515

21. May CR, Eton DT, Boehmer K, et al. Rethinking the patient: using Burden of Treatment Theory to understand the changing dynamics of illness. *BMC Health Services Research* 2014;14:281-81. doi: 10.1186/1472-6963-14-281

22. Carl M, Victor MM, Frances SM. We need minimally disruptive medicine. *BMJ: British Medical Journal* 2009;339(7719):485. doi: 10.1136/bmj.b2803

23. Shippee ND, Allen SV, Leppin AL, et al. Attaining minimally disruptive medicine: Context, challenges and a roadmap for implementation. *Journal of the Royal College of Physicians of Edinburgh* 2015;45(2):118-22. doi: 10.4997/JRCPE.2015.206

24. Tavory I, Timmermans S. Abductive Analysis: Theorizing Qualitative Research: Chicago and London: University of Chicago Press 2014.

25. Gilbrt AW, Jaggi A, May CR, et al. What is the work of being a patient when using communication technology to access healthcare in an orthopaedic setting? A systematic review and meta-synthesis of qualitative studies. PROSPERO CRD42018100896. 2018. http://www.crd.york.ac.uk/PROSPERO/display_record.php?ID=CRD42018100896 (Accessed 3rd October 2019)

26. Critical Appraisal Skills Programme. CASP Qualitative Checklist. 2018 https://casp-uk.net/wp-content/uploads/2018/01/CASP-Qualitative-Checklist-2018.pdf (accessed 18th June 2019).

27. Ryan M, Gerard K, Amaya-Amaya Me. Using Discrete Choice Experiments to Value Health and Health Care: Economics of Non-Market Goods and Resources series, vol. 11. Dordrecht: Springer 2008.

For peer review only - http://bmjopen.bmj.com/site/about/guidelines.xhtml

Figure 1: Overview of the CONNECT PROJECT

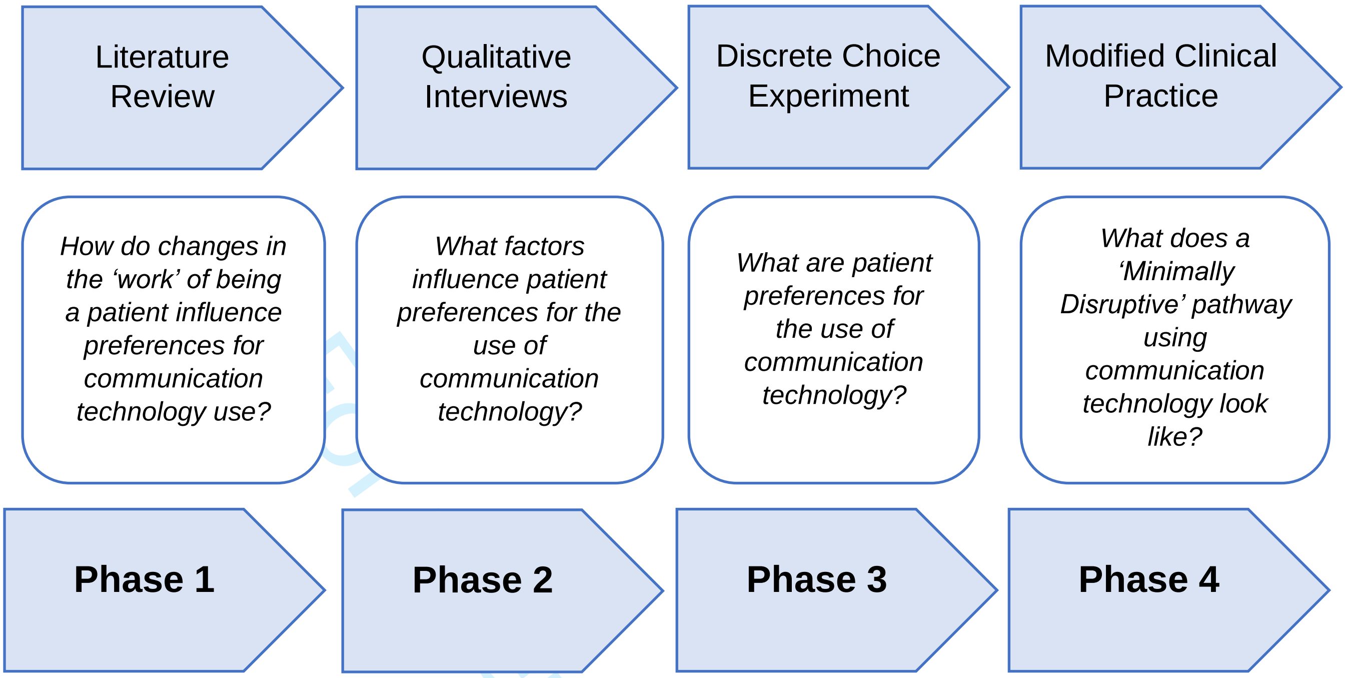

For peer review only

PRISMA-P (Preferred Reporting Items for Systematic review and Meta-Analysis Protocols) 2015 checklist: recommended items to address in a systematic review protocol*

**ADMINISTRATIVE INFORMATION**			
**Title**			**Page**
**Identification**	1a	Identify the report as a protocol of a systematic review	5
**Update**	1b	If the protocol is for an update of a previous systematic review, identify as such	n/a
**Registration**	2	If registered, provide the name of the registry (e.g., PROSPERO) and registration number	2
**Authors**			
**Contact**	3a	Provide name, institutional affiliation, and e-mail address of all protocol authors; provide physical mailing address of corresponding author	1
**Contributions**	3b	Describe contributions of protocol authors and identify the guarantor of the review	11
**Amendments**	4	If the protocol represents an amendment of a previously completed or published protocol, identify as such and list changes; otherwise, state plan for documenting important protocol amendments	n/a
**Support**			
**Sources**	5a	Indicate sources of financial or other support for the review	11
**Sponsor**	5b	Provide name for the review funder and/or sponsor	11
**Role of sponsor/funder**	5c	Describe roles of funder(s), sponsor(s), and/or institution(s), if any, in developing the protocol	n/a
**INTRODUCTION**			
**Rationale**	6	Describe the rationale for the review in the context of what is already known	5
**Objectives**	7	Provide an explicit statement of the question(s) the review will address with reference to participants, interventions, comparators, and outcomes (PICO)	6
**METHODS**			
**Eligibility criteria**	8	Specify the study characteristics (e.g., PICO, study design, setting, time frame) and report characteristics (e.g., years considered, language, publication status) to be used as criteria for eligibility for the review	6
**Information sources**	9	Describe all intended information sources (e.g., electronic databases, contact with study authors, trial registers, or other grey literature sources) with planned dates of coverage	6
**Search strategy**	10	Present draft of search strategy to be used for at least one electronic database, including planned limits, such that it could be repeated	supp
**Study records**			
**Data management**	11a	Describe the mechanism(s) that will be used to manage records and data throughout the review	6

For peer review only

**Selection process**	11b	State the process that will be used for selecting studies (e.g., two independent reviewers) through each phase of the review (i.e., screening, eligibility, and inclusion in meta-analysis)	6
**Data collection process**	11c	Describe planned method of extracting data from reports (e.g., piloting forms, done independently, in duplicate), any processes for obtaining and confirming data from investigators	6
**Data items**	12	List and define all variables for which data will be sought (e.g., PICO items, funding sources), any pre-planned data assumptions and simplifications	N/A
**Outcomes and prioritization**	13	List and define all outcomes for which data will be sought, including prioritization of main and additional outcomes, with rationale	N/A
**Risk of bias in individual studies**	14	Describe anticipated methods for assessing risk of bias of individual studies, including whether this will be done at the outcome or study level, or both; state how this information will be used in data synthesis	6
**Data**			
**Synthesis**	15a	Describe criteria under which study data will be quantitatively synthesized	6
	15b	If data are appropriate for quantitative synthesis, describe planned summary measures, methods of handling data, and methods of combining data from studies, including any planned exploration of consistency (e.g., *I*2, Kendall's tau)	6
	15c	Describe any proposed additional analyses (e.g., sensitivity or subgroup analyses, meta-regression)	N/A
	15d	If quantitative synthesis is not appropriate, describe the type of summary planned	N/A
**Meta-bias(es)**	16	Specify any planned assessment of meta-bias(es) (e.g., publication bias across studies, selective reporting within studies)	N/A
**Confidence in cumulative evidence**	17	Describe how the strength of the body of evidence will be assessed (e.g., GRADE)	N/A

* It is strongly recommended that this checklist be read in conjunction with the PRISMA-P Explanation and Elaboration (cite when available) for important clarification on the items. Amendments to a review protocol should be tracked and dated. The copyright for PRISMA-P (including checklist) is held by the PRISMA-P Group and is distributed under a Creative Commons Attribution Licence 4.0.

From: Shamseer L, Moher D, Clarke M, Ghersi D, Liberati A, Petticrew M, Shekelle P, Stewart L, PRISMA-P Group. Preferred reporting items for systematic review and meta-analysis protocols (PRISMA-P) 2015: elaboration and explanation. BMJ. 2015 Jan 2;349(jan02 1):g7647.

For peer review only

The CONNECT Project Phase 1 Search Strategy

Searches

English Language studies will be searched for with no date restrictions. The following databases will be searched using a combination of keywords and database specific subject headings: MEDLINE, AMED, CINAHL, PsycINFO and Scopus. Forward and backwards reference screening of identified papers and relevant systematic reviews will be completed using Web of Science.

Types of study to be included

Qualitative studies or studies with a qualitative component (use of interviews or focus groups)

Condition or domain being studied

Orthopaedics and musculoskeletal pathologies

Participants/population

Inclusion:
• Full text academic papers.
• Patients with an orthopaedic / musculosketal problem
• Studies reporting patients accessing clinical care (assessment / rehabilitation) through the use of real time communication technology (eg telephone, videoconferencing) in an orthopaedic / musculoskeletal setting.
• Qualitative studies or studies with a qualitative component (use of interviews or focus groups)

Exclusion:
• Conference abstracts
• Participants without an orthopaedic / musculoskeletal complaint
• Quantitative studies
• Studies not reporting patient viewpoints

Intervention(s), exposure(s)

Inclusion: Participants accessing clinical care (assessment / rehabilitation) through the use of communication technology in real time (eg telephone, videoconferencing) in an orthopaedic / musculoskeletal setting.

Comparator(s)/control

N/A

Context

Studies will be included providing they report the perspectives of patients regarding access to musculoskeletal / orthopaedic assessment / rehabilitation. Studies may also contain perspectives of clinicians in addition to the viewpoints of patients.

Main outcome(s)

Patient experience and workload of using communication technology to access musculoskeletal / orthopaedic assessment / rehabilitation.

Timing and effect measures

Not applicable.

Additional outcome(s)

None

Timing and effect measures

None

Data extraction (selection and coding)

Two researchers will independently identify full text papers meeting the inclusion criteria. Discrepancies arising regarding selection will be resolved through discussion with a third researcher.

Risk of bias (quality) assessment

Two researchers will independently screen the full text included studies for risk of bias. The Critical Appraisal Skills Programme Qualitative Tool will be used. Discrepancies arising regarding risk of bias will be resolved through discussion with a third researcher.

Strategy for data synthesis

All studies included for full text review will be analysed. Data extracted from the Methods, Results, Discussion and Conclusion sections will be analysed by using an attribution analysis. A taxonomy of attributions will be thematically analysed using NVIVO software in accordance with Normalisation Process Theory.

Search Terms

Database	Search Term
MEDLINE	(communication technology OR e-health OR telecare OR telemedicine OR telehealth OR telemonitoring OR videoconferencing OR real time videoconferencing OR real time 1:1 videoconferencing OR telecommunication OR virtual OR (MH "telemedicine") OR (MH "remote consultation") OR (MH "videoconferencing")) AND (Focus group OR Focus groups OR observation OR Ethnography OR Ethnographic OR Phenomenology OR Phenomenological OR Lived experience OR Grounded theory OR Thematic analysis OR Conversation analysis

	OR Framework analysis OR (MH "Qualitative Research")) AND (physio OR physiotherapy OR physical therapy OR rehab OR rehabilitation)
AMED	(communication technology OR e-health OR telecare OR telemedicine OR telehealth OR telemonitoring OR videoconferencing OR real time videoconferencing OR real time 1:1 videoconferencing OR telecommunication OR virtual OR (SU (telemedicine) OR (SU (technology medical))) AND (Focus group OR Focus groups OR observation OR Ethnography OR Ethnographic OR Phenomenology OR Phenomenological OR Lived experience OR Grounded theory OR Thematic analysis OR Conversation analysis OR Framework analysis) AND (physio OR physiotherapy OR physical therapy OR rehab OR rehabilitation)
CINAHL	(communication technology OR e-health OR telecare OR telemedicine OR telehealth OR telemonitoring OR videoconferencing OR real time videoconferencing OR real time 1:1 videoconferencing OR telecommunication OR virtual OR (MH “Videoconferencing”) OR (MH “Remote Consultation”)) AND (Focus group OR Focus groups OR observation OR Ethnography OR Ethnographic OR Phenomenology OR Phenomenological OR Lived experience OR Grounded theory OR Thematic analysis OR Conversation analysis OR Framework analysis OR (MH “qualitative studies”)) AND (physio OR physiotherapy OR physical therapy OR rehab OR rehabilitation)
PsychINFO	(communication technology OR e-health OR telecare OR telemedicine OR telehealth OR telemonitoring OR videoconferencing OR real time videoconferencing OR real time 1:1 videoconferencing OR telecommunication OR virtual OR (DE "Telemedicine")) AND (Focus group OR Focus groups OR observation OR Ethnography OR Ethnographic OR Phenomenology OR Phenomenological OR Lived experience OR Grounded theory OR Thematic analysis OR Conversation analysis OR Framework analysis OR DE "Qualitative Research")) AND (physio OR physiotherapy OR physical therapy OR rehab OR rehabilitation)

SCOPUS	1. (TITLE-ABS-KEY (“communication technology” OR e-health OR telecare OR telemedicine OR telehealth OR telemonitoring OR videoconferencing OR “real time videoconferencing” OR “real time 1:1 videoconferencing” OR telecommunication OR virtual))<br>2. (TITLE-ABS-KEY (“Focus group” OR “Focus groups” OR observation OR Ethnography OR Ethnographic OR Phenomenology OR Phenomenological OR “Lived experience” OR “Grounded theory” OR “Thematic analysis” OR “Conversation analysis” OR “Framework analysis”))<br>3. (TITLE-ABS-KEY (physio OR physiotherapy OR physical therapy OR rehab OR rehabilitation)<br>4. #1 AND #2 AND #3

For peer review only

Standards for Reporting Qualitative Research (SRQR)*

http://www.equator-network.org/reporting-guidelines/srqr/

Title and abstract

	Page/line no(s).
**Title** - Concise description of the nature and topic of the study Identifying the study as qualitative or indicating the approach (e.g., ethnography, grounded theory) or data collection methods (e.g., interview, focus group) is recommended	1,7
**Abstract** - Summary of key elements of the study using the abstract format of the intended publication; typically includes background, purpose, methods, results, and conclusions	2,7

Introduction

**Problem formulation** - Description and significance of the problem/phenomenon studied; review of relevant theory and empirical work; problem statement	4,5
**Purpose or research question** - Purpose of the study and specific objectives or questions	7

Methods

**Qualitative approach and research paradigm** - Qualitative approach (e.g., ethnography, grounded theory, case study, phenomenology, narrative research) and guiding theory if appropriate; identifying the research paradigm (e.g., postpositivist, constructivist/ interpretivist) is also recommended; rationale**	5,7
**Researcher characteristics and reflexivity** - Researchers' characteristics that may influence the research, including personal attributes, qualifications/experience, relationship with participants, assumptions, and/or presuppositions; potential or actual interaction between researchers' characteristics and the research questions, approach, methods, results, and/or transferability	8
**Context** - Setting/site and salient contextual factors; rationale**	7
**Sampling strategy** - How and why research participants, documents, or events were selected; criteria for deciding when no further sampling was necessary (e.g., sampling saturation); rationale**	7
**Ethical issues pertaining to human subjects** - Documentation of approval by an appropriate ethics review board and participant consent, or explanation for lack thereof; other confidentiality and data security issues	7
**Data collection methods** - Types of data collected; details of data collection procedures including (as appropriate) start and stop dates of data collection and analysis, iterative process, triangulation of sources/methods, and modification of procedures in response to evolving study findings; rationale**	8

For peer review only - http://bmjopen.bmj.com/site/about/guidelines.xhtml

**Data collection instruments and technologies** - Description of instruments (e.g., interview guides, questionnaires) and devices (e.g., audio recorders) used for data collection; if/how the instrument(s) changed over the course of the study	Supp
**Units of study** - Number and relevant characteristics of participants, documents, or events included in the study; level of participation (could be reported in results)	7
**Data processing** - Methods for processing data prior to and during analysis, including transcription, data entry, data management and security, verification of data integrity, data coding, and anonymization/de-identification of excerpts	8
**Data analysis** - Process by which inferences, themes, etc., were identified and developed, including the researchers involved in data analysis; usually references a specific paradigm or approach; rationale**	8
**Techniques to enhance trustworthiness** - Techniques to enhance trustworthiness and credibility of data analysis (e.g., member checking, audit trail, triangulation); rationale**	8

Results/findings

**Synthesis and interpretation** - Main findings (e.g., interpretations, inferences, and themes); might include development of a theory or model, or integration with prior research or theory	n/a
**Links to empirical data** - Evidence (e.g., quotes, field notes, text excerpts, photographs) to substantiate analytic findings	n/a

Discussion

**Integration with prior work, implications, transferability, and contribution(s) to the field -** Short summary of main findings; explanation of how findings and conclusions connect to, support, elaborate on, or challenge conclusions of earlier scholarship; discussion of scope of application/generalizability; identification of unique contribution(s) to scholarship in a discipline or field	n/a
**Limitations** - Trustworthiness and limitations of findings	n/a

Other

**Conflicts of interest** - Potential sources of influence or perceived influence on study conduct and conclusions; how these were managed	n/a
**Funding** - Sources of funding and other support; role of funders in data collection, interpretation, and reporting	11

*The authors created the SRQR by searching the literature to identify guidelines, reporting standards, and critical appraisal criteria for qualitative research; reviewing the reference lists of retrieved sources; and contacting experts to gain feedback. The SRQR aims to improve the transparency of all aspects of qualitative research by providing clear standards for reporting qualitative research.

For peer review only

**The rationale should briefly discuss the justification for choosing that theory, approach, method, or technique rather than other options available, the assumptions and limitations implicit in those choices, and how those choices influence study conclusions and transferability. As appropriate, the rationale for several items might be discussed together.

Reference:

O'Brien BC, Harris IB, Beckman TJ, Reed DA, Cook DA. **Standards for reporting qualitative research: a synthesis of recommendations.** *Academic Medicine*, Vol. 89, No. 9 / Sept 2014 DOI: 10.1097/ACM.0000000000000388

For peer review only
