# Supplementary material for: Protocol for the CONNECT project: a mixed methods study investigating patient preferences for communication technology use in orthopaedic rehabilitation consultations
Source: BMJ Open. 2019 Dec 11;9(12):e035210. doi: 10.1136/bmjopen-2019-035210 (PMC6924859; doi:10.1136/bmjopen-2019-035210)
Supplement: Reviewer comments [file bmjopen-2019-035210.reviewer_comments.pdf]

PEER REVIEW HISTORY

BMJ Open publishes all reviews undertaken for accepted manuscripts. Reviewers are asked to complete a checklist review form (**http://bmjopen.bmj.com/site/about/resources/checklist.pdf**) and are provided with free text boxes to elaborate on their assessment. These free text comments are reproduced below.

ARTICLE DETAILS

**TITLE (PROVISIONAL)**	Protocol for the CONNECT Project: a mixed methods study investigating patient preferences for communication technology use in orthopaedic rehabilitation consultations
**AUTHORS**	Gilbert, Anthony William; Jones, Jeremy; Stokes, Maria; Mentzakis, Emmanouil; May, Carl R

This article was not externally reviewed at BMJ Open. Protocols that have been independently assessed prior to submission to BMJ Open are usually fast-tracked to publication on the grounds that further substantial changes will not be possible. This independent assessment will usually be external, independent review for both a substantial grant award from a non-commercial or government funder and ethics approval.
